# Neuroprotective effects of strength training in a neuroinflammatory animal model

**DOI:** 10.1186/s12868-022-00708-w

**Published:** 2022-04-11

**Authors:** Elizama de Gregório, Gabriela Cristiane Mendes, Lincon Bordignon Somensi, Cassio Geremia Freire, Luiza Freitas Lopes, Karine Ramires Lima, Guilherme Salgado Carrazoni, Ben-Hur Souto Neves, Steffanie Severo Picua, Luisa Mota da Silva, Pamela Billig Mello-Carpes, Juliana Sartori Bonini, Weber Claudio da Silva

**Affiliations:** 1 Department of Medicine, Postgraduate Program in Development and Society (PPGDS), University of Alto Vale Do Rio Do Peixe (UNIARP), Caçador, SC Brazil; 2 Physiology Research Group, Stress, Memory and Behavior Lab, Federal University of Pampa, Uruguaiana, RS Brazil; 3 Postgraduate Program in Pharmaceutical Sciences (PPGCF), Center for Chemical-Pharmaceutical Research (NIQFAR), University of Vale Do Itajaí (UNIVALI), Itajaí, SC Brazil; 4 Laboratory of Neuropsychopharmacology, Pharmacy, Department, State University of Midwest of Paraná, Guarapuava, PR Brazil; 5 Postgraduate Program in Physiology, Physiology Department, Federal University of Rio Grande Do Sul (UFRGS), Porto Alegre, RS Brazil

**Keywords:** Neuroinflammation, Strength training, Oxidative stress, Memory

## Abstract

**Background:**

The preventive role of muscular strength on diminishing neuroinflammation is yet unknown. In this study, the role of the prophylactic muscular strength exercise was investigated in order to verify whether it would diminish cognitive alterations and modify the antioxidant intracellular scenery in an animal neuroinflammatory model in of the CA1 region of the hippocampus.

**Methods:**

The animals received muscular strength training (SE) three times a week for eight weeks. Subsequently, the stereotaxic surgery was performed with an intra-hippocampal infusion of either saline solution (SAL) or lipopolysaccharide (LPS). Next, we performed the behavioral tests: object recognition and social recognition. Then, the animals were euthanized, and their hippocampus and prefrontal cortex were collected. In another moment, we performed the dosage of the antioxidant activity and histological analysis.

**Results:**

The results showed that the muscular strength exercises could show a beneficial prophylactic effect in the cognitive deficiencies caused by acute neuroinflammation. Regarding oxidative stress, there was an increase in catalase enzyme activity (CAT) in the group (SE + LPS) compared to the control groups (p < 0.05). As for the cognitive alterations, there were found in the (SE + LPS) group, diminishing the mnemonic hazard of the discriminative and social memories compared to the control groups (p < 0.05).

**Conclusion:**

We concluded, therefore, that the exercise performed prophylactically presents a protective effect capable of minimizing such mnemonic deficits and increasing catalase enzyme activity in rats that suffered a local neuroinflammatory process in the hippocampus.

**Supplementary Information:**

The online version contains supplementary material available at 10.1186/s12868-022-00708-w.

## Introduction

Neuroinflammation is a complex process that consists of the answer of specialized immune cells in the central nervous system (CNS) in response to a stimulus that affects the basal activity of this tissue [1]. However, because the brain has been considered an immune-privileged organ since peripheral immune cells are unable to cross the blood–brain barriers (BBB), limiting the neuroinflammatory response to the CNS, the interaction with the peripheral immune system is little known [1–3].

Microglia cells have been considered the immune cells on the CNS and express pro and anti-inflammatory factors, promoting brain homeostasis [3]. In its basal physiological activity, microglia express an anti-inflammatory phenotype, and their ramifications go through continuous extension cycles to monitor the environment [2, 4].

However, when detecting a pathological stimulation, such as infections, mechanical trauma, poorly folded proteins, and ischemia [4], microglia quickly modifies its morphology, expressing a reactive phenotype with pro-inflammatory effects, synthesizing and secreting cytokines, such as TNF-α and IL-1β [5], activating nitric oxide synthesis -2 enzyme, cyclooxygenase 2 (COX2) and C–C chemokine ligand 2 (CCL2), which in turn promotes the elevation of the reactive oxygen species (ROS) production [6], aiming to eliminate the aggressive stimulation and repair the tissue damage [1, 7].

It is known that the neuroinflammatory process is a needed mechanism and important resolution of aggressor factors to the SNC [3]. However, when harmful outside (environmental) and/or internal persistent stimulation are recognized by the microglia [8], a system of positive retro-feeding is sustained, over-elevating the production of pro-inflammatory factors, which promotes cell death and atrophy of local neural synapses, inducing symptoms such as cognitive decline, movement alterations and memory loss [4].

The constant activation of the microglia, the increase of the oxidative stress, the reduction of the neurotrophic support, the alteration of the metabolism of neurotransmitters, and the rupture of the brain-blood barrier are described in the literature as mechanisms underlying neuropsychiatric disorders, including Alzheimer's disease [6, 9], Parkinson disease and depression [5].

It is foreseen that neuroinflammatory/neurodegenerative diseases will be the biggest health concern of this century and the second main cause of death by 2050 [10]. From this perspective, several studies are performed to develop therapeutic and preventive strategies that may mold the neuroinflammatory process. The practice of physical exercise, a non-pharmacological action, has shown to be promising in the search to reach such goals [11], and has been proposed as an antioxidant and natural anti-inflammatory strategy, which stimulates the increase of hippocampal neuroplasticity [3, 12], hippocampal neurogenesis and cell proliferation [13], as well as it increases the synthesis of growth factors such as the brain-derived neurotrophic factor (BDNF) [14, 15], insulin-like growth factor 1 (IGF-1) [16] and the production of enzymatic and non-enzymatic antioxidants [3].

All such benefits have a direct action on the symptomatology of patients with neuroinflammatory diseases, diminishing the cognitive decline, especially memory loss [3, 11]. However, most relevant studies are focused on the benefits of aerobic exercises. Few studies approach the influence of muscular strength training, especially when practiced prophylactically, to prevent a possible progression of neuroinflammation, how such effects are modulated, and what the best strategy of physical training, is regarding intensity, training, and recovery period.

In this context, we investigated the hypothesis about the neuroprotective effect of muscular strength training, practiced prophylactically, in a neuroinflammation-induced by l*ipopolysaccharide* (LPS) in rodents. For such, we analyzed the discriminative, aversive, and social memory, histopathological features, and the activity of antioxidant enzymes in the hippocampus and in the prefrontal cortex of rats which were submitted to the hippocampus intra-CA1 infusion of LPS after eight weeks of physical training.

## Methods

### Animals and experimental design

Male *Wistar* rats of approximately two months old and weight of 250 – 350 g were used. The animals were conditioned in controlled room temperature at 22 ºC with light/dark cycles of 12 h and kept in appropriate boxes with capacity for 5 animals, covered with shaving, which was switched every two days. They received water and food ad libitum. The maximum concern was deliberated with the purpose of minimizing the suffering of the animals and reducing the number of animals to be used in the research.

The animals were divided into six groups (n = 10) and were divided into control groups: group naïve, group submitted to the intra-hippocampal infusion of saline, group submitted to the hippocampus infusion of LPS; and groups which practiced muscular strength exercises: a group which performed only muscular strength exercises (CT), a group which performed muscular strength exercises and was submitted to the infra-hippocampal infusion of saline (exercise + saline) (n = 10), and a group that performed muscular strength exercise and was submitted to the intra-hippocampal infusion of LPS (exercise + LPS) (n = 10).

The animals were submitted to eight weeks of training three times a week. After the final day of training, all animals performed the muscular strength testing by gripping through the Grip Strength equipment. Then, the surgery was performed to induce acute neuroinflammation through LPS, which was infused in the CA1 region of the hippocampus. The post-operative period was five days, followed by the behavioral tests in the following sequence: open field, object recognition, social recognition, plus maze and hot plate. At last, the animals were euthanized. Then, the heart of each animal was removed to access its mass, whereas the hippocampus, and the prefrontal cortex were removed to analyze the activity of antioxidant enzymes. As per the Additional file 2: Figure S1.

### Ethics approval

All the experiments were according to the rules of the Ethics Committee in the Use of Animals—Federal University of Pampa (CEUA/UNIPAMPA) (report #046/2017). the study is reported in accordance with ARRIVE guidelines.

### Strength exercise protocol

Muscular strength training was performed using a personalized vertical scale made of wood and iron (1.1 × 0.18 m, 2 cm grip, an inclination of 80°) with a wooden box (20 × 20 × 20 cm) put in the upper part of the stairs. Initially, the animals were familiarized with the exercise, performing four climbing attempts per day for three days. Once they were able to climb to the wooden box, they could rest inside the wooden box for 120 s. The strength training started a week after the familiarization. In the first week, the load corresponding to 50% of the rat's body mass was fixed at the base of the animal's tail. In the second week of training, the maximum load test was performed to determine a load of exercise for each animal individually. It was added 30 g to each repetition of the next climb until the rat was not able to finish the exercise. The heavier load transported successfully was considered the maximum load. The maximum load was determined on the first day of each week of training. The training sessions consisted of eight climbs in the stairs with two repetitions for each load of 50%, 75%, 90%, and 100% of the individual result of the maximum load, resulting in 8 climbs with a rest interval of 1 min between the repetitions, with three training sessions per week (one day for the maximum load and two days for the training sessions), during eight weeks [17].

### Grip strength meter

After the final day of training, all animals performed the muscular strength test by gripping through Grip Strength equipment. The grip gauge was positioned horizontally facing the equipment. The animals were put in the metal grid and, next, were pulled backward in the horizontal plane by the tail. The forces applied to the grid through the animal's legs were measured in grams. Immediately before losing the adherence, the maximum tension was recorded. Three tests were performed, and as a result, a mean value was obtained [18].

### LPS administration

The animals were submitted to stereotaxic surgery. Subsequently, the intra-hippocampal bilateral administration (CA1 region) administration of vehicle (saline solution) or LPS (from *Escherichia coli* 055:B5; Sigma) dissolved in PBS in the concentration of 10 mg/ml and administrated in the dose of 40 μg/side, through the infusion of 4 μL/side during 10 min. The stereotaxic surgery coordinates were adapted from the Paxinos & Watson Anatomic Atlas, being as follows: antero-posterior (AP) = − 4.2 mm; mid-lateral (MD) =  ± 3.0 mm; dorsum-ventral (DV) = − 3.0 mm; lateral tilting (LT) = 0° starting from the Bregma point. The LPS infusion was performed using a Hamilton (5 µl) syringe. The procedures were performed with previously anesthetized animals with 75 mg/kg of ketamine and 10 mg/kg of xylazine through the intra-peritoneal route. After the surgery, the rats were put in housing boxes under smooth heating to avoid hypothermia.

### Object recognition memory test

From the first to the fourth day of the experiment of such task, regarding the habituation period, the animal was put in the left superior corners of the device, a 50 cm × 50 cm × 50 cm box, made of compensated, acrylic transparent polyvinyl chloride, and then left it there for 5 min, without a single object inside the box, so that the animal could get used to the environment.

During the training session, one day after the last day of the habituation day, the animal was once again put in the box with two equal objects, A and B, located right at the center of the box. The animal was left there for 5 min to explore the environment freely. The exploration time of each object was clocked for posterior evaluation. After 3 h, the short-term memory was tested. The animal was replaced in the box with object A and object C (replacing the previous object B) in 5 min for free exploration. The long-term memory duration was tested 24 h after the previous test, in which the animal was once again placed inside the box, with objects A and D (different from objects A, B, and C), and the animal had 5 min for free exploration [19].

### Social recognition memory test

This task is an adaptation of the social interaction test proposed by Kaidanovich-Beilin et al*.,* [20]. The task was performed in 3 days. First, the animals were placed in a habituation field (the same size and characteristics described in the previously described object recognition task) with two small cages for 20 min for free exploration. On the following day, the training was performed with a juvenile rat in one of the cages for one hour of free exploration. At the same time, one cage was left empty. After 24 h, the test was performed when the same rat of the training (that is, the rat that became familiar) and a new rat were put for exploration for 5 min. The time exploring the new rat and the familiar rat was registered. Exploring the animal was defined as smelling or touching the cages with the nose or front legs [21].

### Open-field test

To evaluate the locomotor ability of the animals, they were put for 5 min in the open-field arena. The apparatus has the same size and characteristics previously described in the object recognition task. The experiments were held in a low sound room under low-intensity lighting. Each rat was placed in the center of the open field and the number of squares crossed and rearing were registered [22].

### Elevated plus-maze

To evaluate the state of anxiety of the animals, they were put in an elevated crossway maze (ECM), and the number of entries and the time spent in the open and close arms were registered during a 5-min session [23].

### Hot plate

To evaluate the nociceptive response and the sensitivity of the animal's legs, each of them was placed in a device with a warm metal sheet of paper (55 ± 0,5 °C) and the time until the animal showed reaction to the thermal stimulation by raising or licking one's leg was determined [24].

### Euthanasia

The animals were sacrificed by physical method, through decapitation by guillotine. The entire procedure was based on the guidelines written by the national council for control of animal experimentation (CONCEA), which aims to establish procedures that evoke minor pain and suffering to the animal.

### Heart mass

After all the behavioral tasks, the animals were euthanized, and then the collection of the hippocampus, prefrontal cortex, and heart mass were performed. For the heat mass collection, the open chest procedure was performed, and blood was drawn by cardiac puncture, followed by the removal of the heart. After such removal, the heart was washed with saline solution (0.9%) to remove the excess blood. The heart mass was immediately weighed using an analytic scale. The cardiac hypertrophy index was calculated by the reason between heart mass (mg) and body mass (g).

### Histological analysis

After euthanasia, the hippocampus was removed and then fixed in 10% formaldehyde, dehydrated, and embedded in paraffin. Then, Sects. (5 mm) were stained with hematoxylin and eosin (H&E) and analyzed under a microscope. The physical dissection method was used for quantitative analysis of dark neurons recognized by hyper basophilia density and morphological features [25, 26]. All dark neurons present in 400 × 400 µm squares were quantified using ImageJ® software version 1.52c (National Institutes of Health, Bethesda, MD, USA) and visually confirmed using an optical microscope (L3000B, Labor Import®, 400 × magnification). For each rat, three squares were analyzed for the CA1 and dentate gyrus (DG) regions and two squares for the CA3 region. The mean of these values was used as the final value in dark neurons/mm^2^.

### Preparation of the homogenate and protein analysis

Tissue samples of the hippocampus and prefrontal cortex were weighed and homogenized with 200 mM phosphate buffer. This homogenate was used for the quantification of the reduced glutathione (GSH) and lipoperoxides (LOOH) amounts. Further, the homogenate obtained was centrifuged at 10,000 rpm for 20 min. The precipitate was used to the determination of myeloperoxidase enzymes (MPO), whereas the supernatant was used to verify the dismutase superoxide (SOD), catalase (CAT) and S-transferase glutathione (GST) activities.

The protein concentration was measured with a spectrophotometer at 590 nm using Bradford reagent, and bovine albumin (0.012 – 0.100 mg/mL) was used for the standard curve.

### Determination of MPO activity

The obtained precipitate (according to the previous description) was re-suspended with 500 µL of 80 mM potassium phosphate buffer with 0.5% of hexadecyltrimethylammonium bromide. After the homogenization, the samples were once again centrifuged at 12,000 rpm, 20 min at 4 °C of temperature in a high-speed refrigerated microcentrifuge. Aliquots of 60 μl of the supernatant of each sample was added to 200 μL of a reactional solution (100 μL of 80 mM phosphate buffer, 85 μL of 22 mM phosphate buffer, and 15 μL of 0.017% H2O2) in triplicate using a 96-well plate. The reaction was started with the addition of 20 μl of tetramethylbenzidine and incubated for 3 min at 37ºC. After, the reaction was interrupted by adding 30 μL of 1,46 M sodium acetate (pH = 3.0) in each well. The absorbance was read at 620 nm, and the results were expressed as units of optical density (OD)/mg of protein, according to Bradley et al. [27].

### Quantification of the GSH and LOOH levels

As described previously [28, 29], 50 µl of the homogenate was added to 40 µL of 12,5% of trichloroacetic. Next, the material was centrifuged at 9000 rpm during 15 min. After the centrifuge, 20 μL of the supernatant was added to 270 μL of TRIS buffer (pH 8.9) and 10 μL of 5,5'-dithio-bis-[2-nitrobenzoic acid (DTNB). The absorbance was measured after 5 min at 415 nm, and the obtained values were interpolated in a standard deviation curve of GSH (1.25–10.00 μg/mL) and expressed as μg/g of tissue.

### Determination of SOD, CAT, and GST activities

The obtained supernatant (according to the previous description were used to verify the SOD activity, and it was based on the capacity of the SOD in inhibiting the auto-oxidation of the pyrogallol. In a conic tube, the following were added: 442,5 μL of Tris -EDTA buffer and 20 μL of the sample. After agitation, 25 μLof 1 mM pyrogallol was added and incubated. Then, they were centrifuged, and 300 μL of the supernatant was pipetted in a microplate for the reading in the spectrophotometer at 205 nm. The results were compared with the control group being such value equal to 100%. The amount of protein that inhibits the reaction in 50% (IC 50) equals one unit (U) of SOD. The results were expressed in U of SOD/mg of protein [30].

The supernatant was also used to verify the participation of the catalase enzyme (CAT). In a 96-well microplate 10 μL of the sample was placed, added to 290 go of the reaction broth (5 mM Tris/EDTA buffer, pH 8.0, plus 30% H2O2 and ultra-pure water) [31]. After, the decrease in the absorbance was monitored at 240 nm. The results were expressed in µmol of H_2_O_2_ consumed/min/g of tissue.

The supernatant was used to verify the activity of the glutathione S-transferase enzyme. In this assay, 50 μL of the supernatant and 250 μL of the reaction broth (0,1 M phosphate buffer, 3 mM 1-Chloro-2,4-dinitrobenzene and 3 mM GSH) were added in a 96-well microplate. Then, the increase in the absorbance was monitored at 340 nm, and the results were expressed in μmol/min/mg of protein [32].

### Data analysis

The latency and the time percentage of the behavioral experiments and the mean numbers obtained in the biochemical experiments were compared between the different groups through the parametric Student test (two groups) or ANOVA (more than two groups) with the post-hoc Newman-Keuls test for multiple comparisons, or Dunnett’s test for the comparisons with the control group. P values < 0.05 were considered statistically significant.

## Results

Data from the bodyweight were expressed in grams. All animals gained weight throughout the experiment. However, there was no significant difference regarding weight gain among the different groups: CT (442.7 ± 20.43), saline + exercise (419.8 ± 16.47), LPS + exercise (378.7 ± 14.90), Naive (445.4 ± 14.88), saline (429.4 ± 11.37) and LPS (425.7 ± 14.70) (p > 0.05).

The animals submitted to the strength exercise protocol performed the maximum load test once a week, during all the muscular strength training protocols, to evaluate the animal's maximum muscular strength and next prescribe the maximum load that the animal would use during the exercise.

The prophylactic intervention with exercise resulted in progressive muscular strength increase in the animals trained for eight weeks. However, it was not verified significant difference among the trained groups that received LPS/saline or the control group (CT) (Additional file 2: Figure S2).

## Grip strength testing

**Fig. 1:**
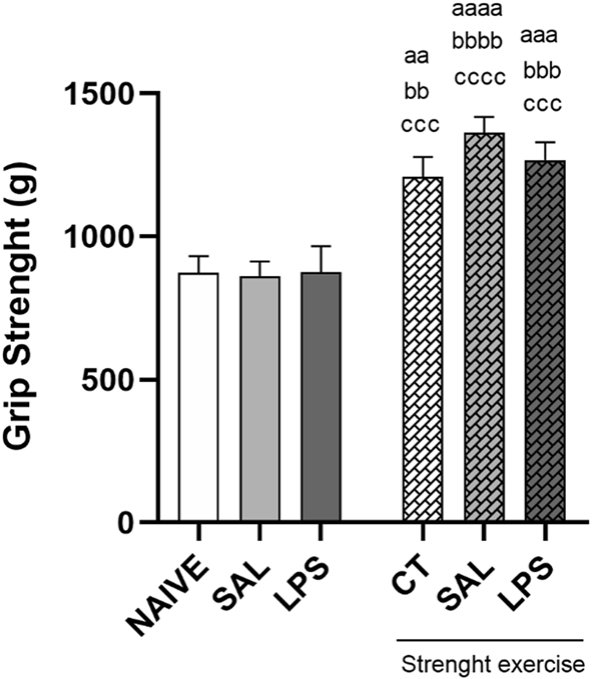
Eight weeks of strength exercise previously to the infusion of intra-CA1 dorsal bilateral infusion of LPS (40 µg/side) induced an increase in the grip strength

After the final day of muscular strength training, the animals were submitted to the grip strength testing through the equipment Grip Strength Meter. The results of the outcome of the test for all the groups are shown in Fig. 1.

The rats were submitted to eight weeks of strength exercise training protocol. As followed, they received an intra-CA1 dorsal bilateral infusion of either saline (SAL) or LPS (40 µg/side). Groups NAIVE and control (CT) were not submitted to the procedure of intra-CA1 infusion. One day after the final day of training, the animals were submitted to the grip strength test (g) through the equipment Grip Strength Meter. Analysis of variance two-way ANOVA (F_(5, 53)_ = 11.92) followed by Newman-Keuls multiple comparison test, aa *P* < 0.01; aaa *P* < 0.001; aaaa *P* < 0.0001, when compared with the group NAIVE. bb *P* < 0.01; bbb *P* < 0.001; bbbb *P* < 0.0001 when compared with the SAL group, when not submitted to exercise. ccc *P* < 0.001; cccc *P* < 0.0001 when compared to the LPS group, when not submitted to exercise.

As expected, the prophylactic intervention with exercise resulted in grip strength increase in the trained animals, demonstrating that the proposed training protocol in the present study was efficient, significantly increasing the muscular strength of the trained animals compared to the animals which were not trained.

## Open field test

During the habituation phase, the animals had their locomotor and exploratory capacity analyzed in the open field task, which preceded that object recognition task. The performance results in such a task for all the groups are shown in Additional file 2: Figure S3.

There were no significant differences among the groups throughout the days in the analyzed parameters analyzed in the open field task. Therefore, neither the resistance exercise nor the neuroinflammation induced by the LPS in the CA1 hippocampus region was able to alter the locomotor and exploratory activity of rats in the open field task regarding the control group.

## Object Recognition Test

**Fig. 2:**
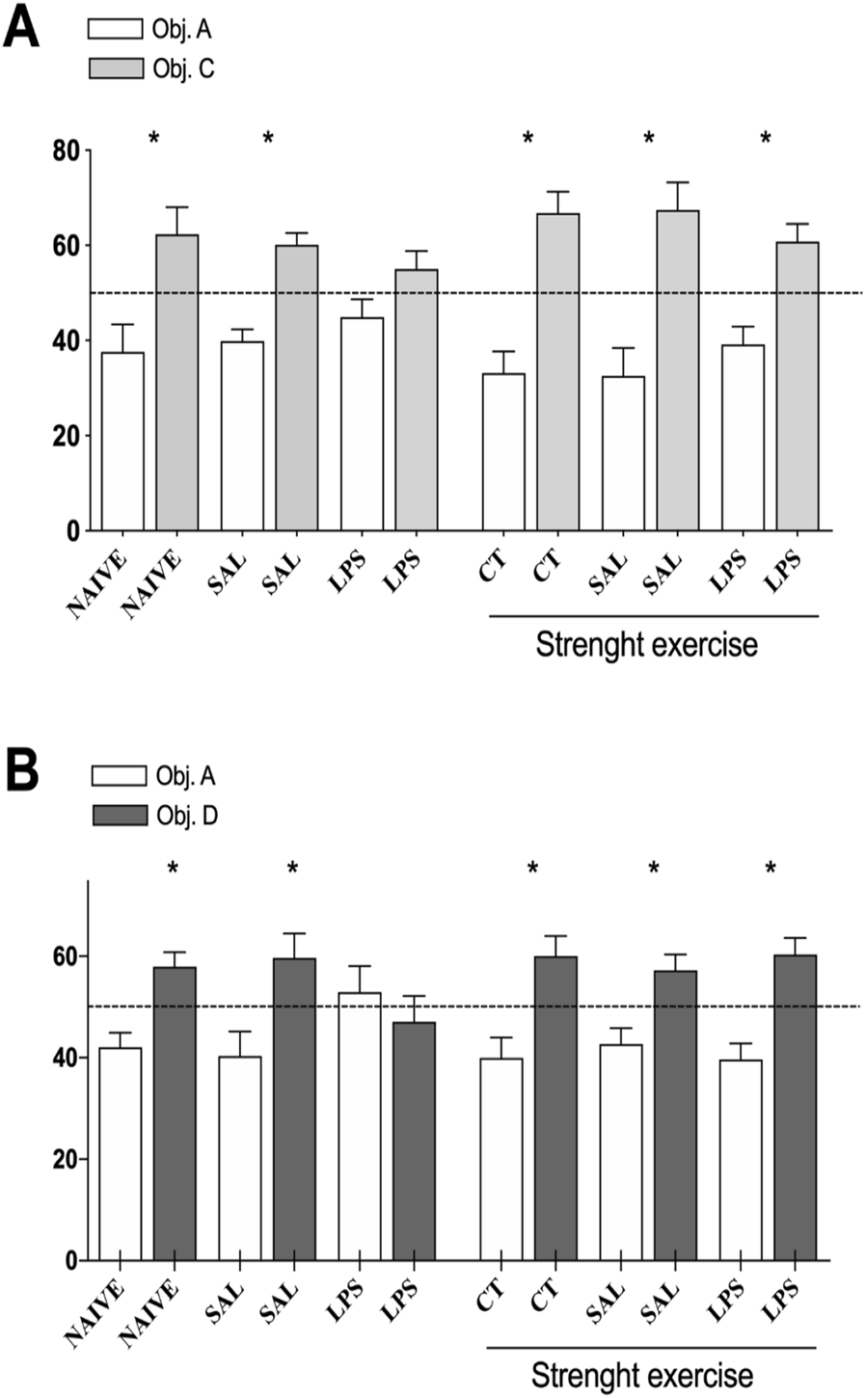
Eight weeks of strength exercise previous to the intra-CA1 dorsal bilateral infusion of LPS (40 µg/side) improves the performance of rats in the retention of the short-term (STM) or recent long-term discriminative memory (LTM) relative to the object recognition task

The animals had their short and long-term discriminative memory analyzed in the object recognition task. The performance results in such a task are shown in Fig. 2.

The rats were submitted to the strength exercise training protocol for eight weeks. As followed, they received an intra-CA1 dorsal bilateral infusion of either saline (SAL) or LPS (40 µg/side). After five days of post-operative recovery, these rats were submitted to several behavioral tasks, including the object recognition task. (A) The test was performed 3 h after the training session with the objects A and B. (B) The second test was performed 24 h later. Data are expressed as mean numbers (± EM) from the exploration percentage of each of the objects presented regarding the total time of the exploration of the objects. * p < 0.05 vs. Theoretical percentage of 50% in Student's t-test. (n = 7–10 per group).

As expected, the locally provoked neuroinflammation in the CA1 hippocampus region harmed the rats' capacity to form and retain discriminative short and long-term duration regarding object recognition task. The prophylactic intervention with strength exercise resulted in a protective effect, which was verified both in the retention of the short-term and recent long-term discriminative memory. Therefore, in this cognitive task, the strength exercise has been shown to have a potential prophylactic mnemonic neuroprotective effect in the face of a local neuroinflammatory effect.

## Social recognition test

**Fig. 3:**
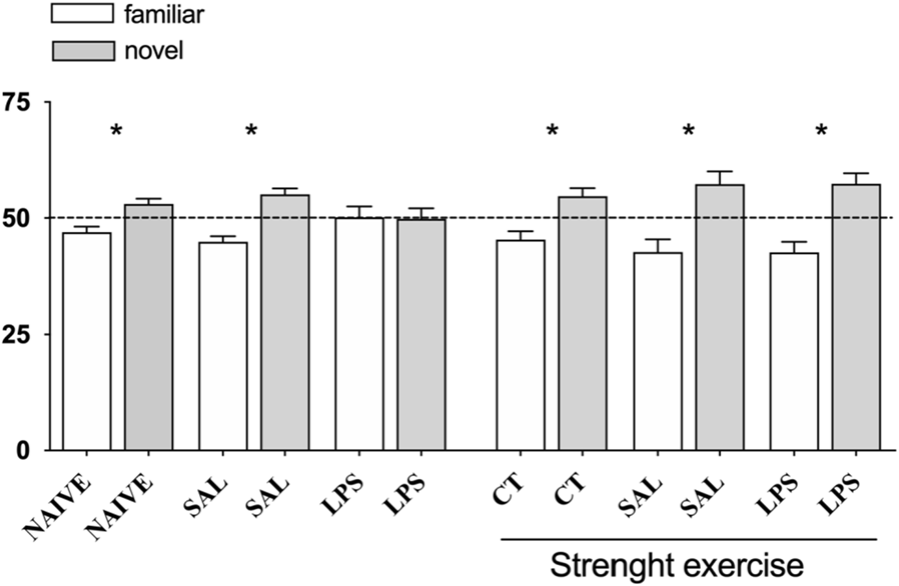
Eight weeks of strength exercise, previously to the intra-CA1 dorsal bilateral infusion of LPS (40 µg/side), improves the performance of rats in the retention of recent long-term social memory (LTM) regarding the task of social recognition

The animals had their retention capacity of short and long duration of social memory analyzed in the social recognition task. The performance results in such a task for all the groups are shown in Fig. 3.

The rats were submitted to eight weeks of training in the strength exercise protocol. As followed, they received an intra-CA1 dorsal infusion of either saline (SAL) or LPS (40 µg/side). The groups NAIVE and control (CT) were not submitted to the intra-CAI infusion procedure. After five days of post-operative recovery, these rats were submitted to several behavioral tasks, including the social recognition task. Test performed 24 h after the training session. Data are expressed as mean numbers (± EM) from the percentage of the exploration of each of the juvenile rats regarding the total time of exploration of such rats. * p < 0.05 vs. Theoretical percentage in 50% in Student t-test. (n = 7–10 per group).

As expected, the locally provoked neuroinflammation in the CA1 hippocampus region harmed the capacity of the rats to form and retain short and long-term social memory related to the social recognition task. The prophylactic intervention with strength exercise resulted in a verified protective effect on the recent long-term social memory retention. Therefore, in this cognitive task, the strength exercise has shown to have prophylactic neuroprotective potential in the face of an acute neuroinflammatory context.

## Plus maze test

The animals had their level of anxiety analyzed in the elevated plus-maze. The performance results in such a task for all groups are shown in Additional file 2: Figure S4.

There were no significant differences among the groups in the analyzed parameters in the elevated plus-maze task. Therefore, neither the strength exercise nor the neuroinflammation induced by the LPS in the CA1 hippocampus region could alter the level of anxiety in the rats in such task regarding the control groups.

## Hot plate test

The animals had their nociception analyzed in the hot plate task. The performance results in such a task for all the groups are shown in Additional file 2: Figure S5.

There was no significant statistical difference among the groups regarding the reaction time to the thermal stimulation by removing and licking the legs. Therefore, neither the strength exercise nor the induced neuroinflammation by the LPS in the CA1 hippocampus region could alter the nociception of the rats in this task regarding the control groups.

## Heart mass

**Fig. 4:**
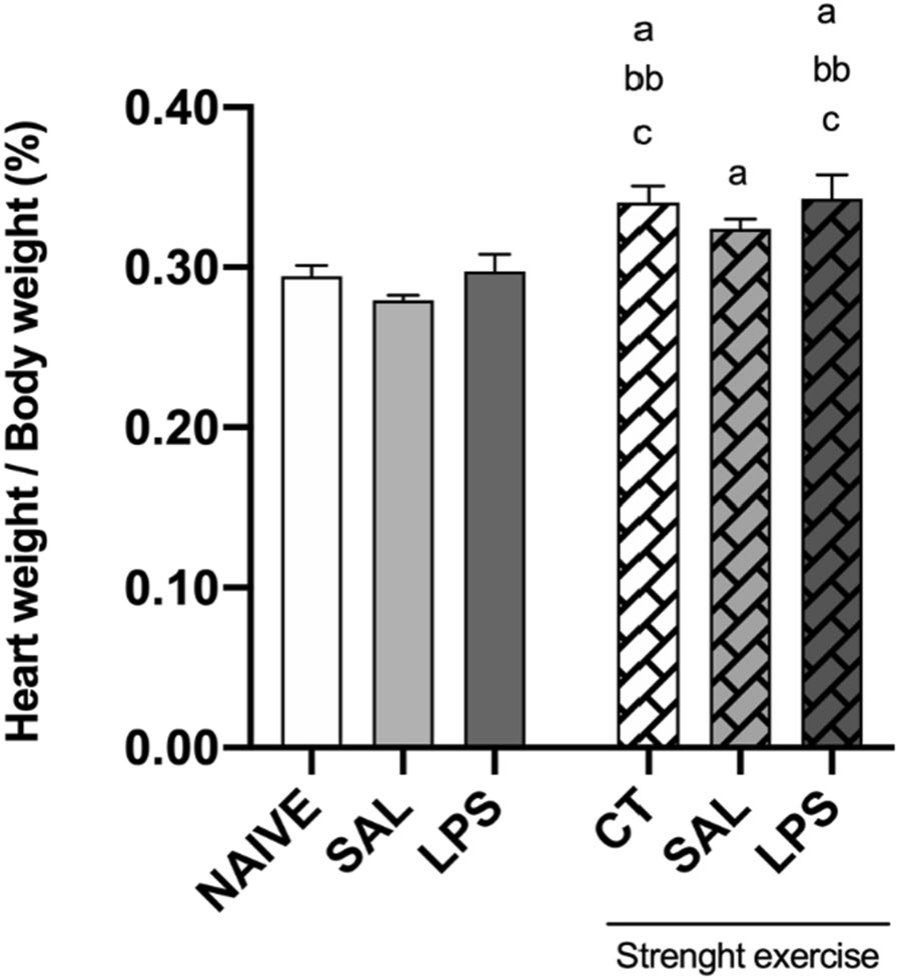
Eight weeks of strength exercise previously to the intra-CA1 dorsal bilateral infusion of (40 µg/side) induces physiological cardiac hypertrophy caused by muscular training

Figure 4 presents the data referring to the heart mass, presented as the reason between the mass of the heart by body mass of the animal, index which determines the occurrence of cardiac hypertrophy. The heart mass/body mass from the group CT, SAL + exercise, and LPS + exercise was significantly higher than the respective groups NAIVE, SAL without exercise, and LPS without exercise.

The rats were submitted to eight weeks of training in the strength exercise protocol. As followed, they received the intra-CA1 dorsal bilateral infusion of saline (SAL) or LPS (40 µg/side). The groups NAIVE and control (CT) were not submitted to the intra-CA1 infusion procedure. After being euthanized, the animals' heart was removed and then weighted. The body mass corrected the heart mass. The following formula was used for such correction: (Total weight of the heart/body weight) × 100. One-way ANOVA (F_(5, 29)_ = 6.282), followed by Newman-Keuls multiple comparison tests, a *P* < 0.05 when compared to the group NAIVE. bb *P* < 0.01 when compared to the group SAL not exercised. c *P* < 0.05, when compared to the group, LPS not exercised.

The prophylactic intervention of muscular strength exercise induced physiological hypertrophy, compensatory/adaptation needed to maintain a cardiac performance in the increased condition of circulatory overload during the animals' training. It is known that intensive and long-lasting physical training induces cardiovascular adaptations, including cardiac hypertrophy, which allows the heart an exceptional physical performance. We noticed, in this study, a statistical difference in the heart mass of the animals which trained, when compared to the ones that did not train, which shows that the heart of the trained animals became adapted to the muscular strength training, showing that the proposed training protocol in the present study was efficient.

## Dark cell count

The rats were subjected to eight weeks of training in the strength exercise protocol. They subsequently received a bilateral intra-CA1 dorsal infusion of saline (SAL) or LPS (40 µg/side). After euthanasia, the hippocampus was removed and then fixed in 10% formaldehyde, dehydrated, and paraffin embedded. Then, Sects. (5 mm) were stained with hematoxylin and eosin (H&E). All dark neurons present in 400 × 400 µm squares were quantified using ImageJ® software version 1.52c (National Institutes of Health, Bethesda, MD, USA) and visually confirmed using an optical microscope (L3000B, Labor Import®, 400 × magnification). One-way ANOVA (F_(3, 11)_ = 4.591), followed by Holm-Sidak post-test, aa P < 0.01.

**Fig. 5:**
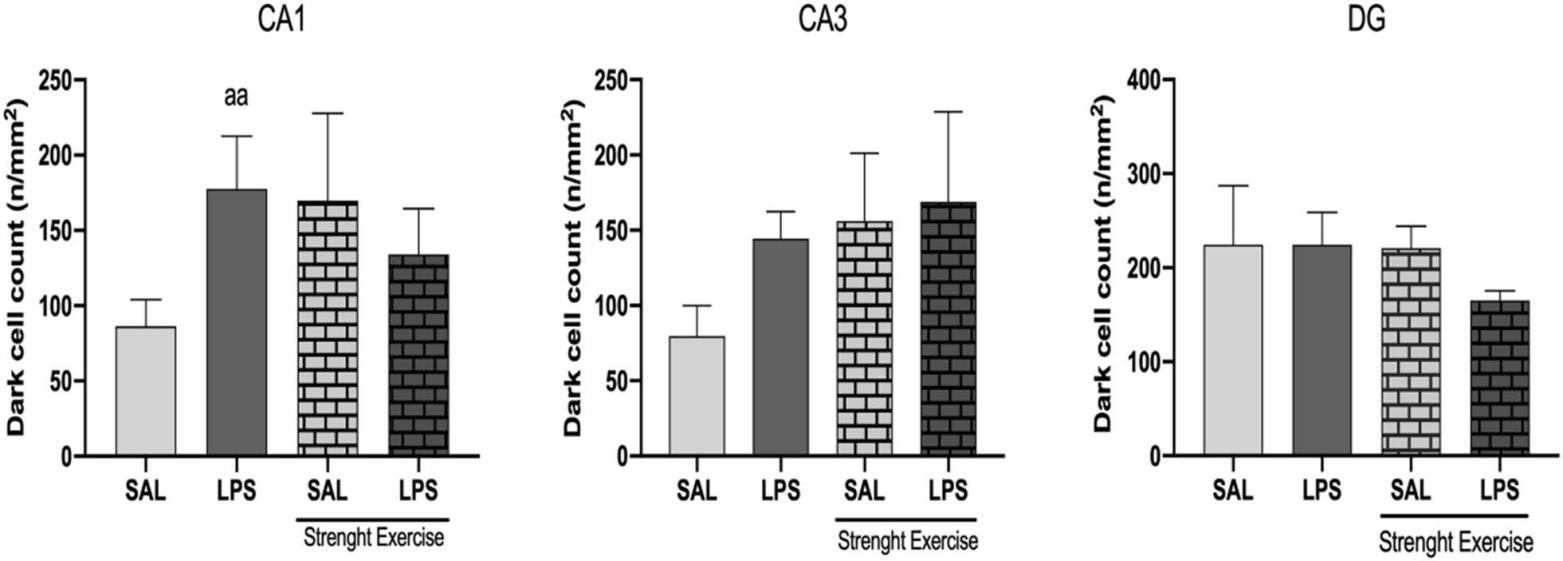
Eight weeks of strength exercise hindered the enhancement of rats' dark neuron count verified after intra-CA1 dorsal bilateral infusion of LPS (40 µg/side) in the hippocampus CA1 region

Figure 5 shows the prophylactic muscle strength exercise intervention did not influence per se the dark cell count recognized by hyperbasophilia density and morphological features (characteristics of a degenerating neuron) in CA1, CA3 and DG regions. We observed that the non-exercised group that received an intra-hippocampal infusion of LPS showed a higher number of dark neurons in the CA1 region when compared to the group that received an intra-hippocampal infusion of saline, but this increase between these groups (higher number of dark neurons in the group infused intra-CA1 with LPS than with SAL) did not occur in the exercised rats. We also observed a tendency for this statistical difference between the non-exercised rats that received either intra-CA1 or saline or LPS infusion in the CA3 region, probably as a matter of proximity. We found no static difference between the groups in the DG region, possibly because of the distances between the CA1 region (which received the LPS infusion) and DG, so LPS did not significantly affect this region. The histological images are in the Additional file 2: Figure S6.

### Effect of strength exercise on the expression of GSH levels and the activity of MPO, SOD, CAT and GST enzymes in the CA1 region of hippocampus from rats after infusion of LPS in the CA1 region of the hippocampus

**Fig. 6:**
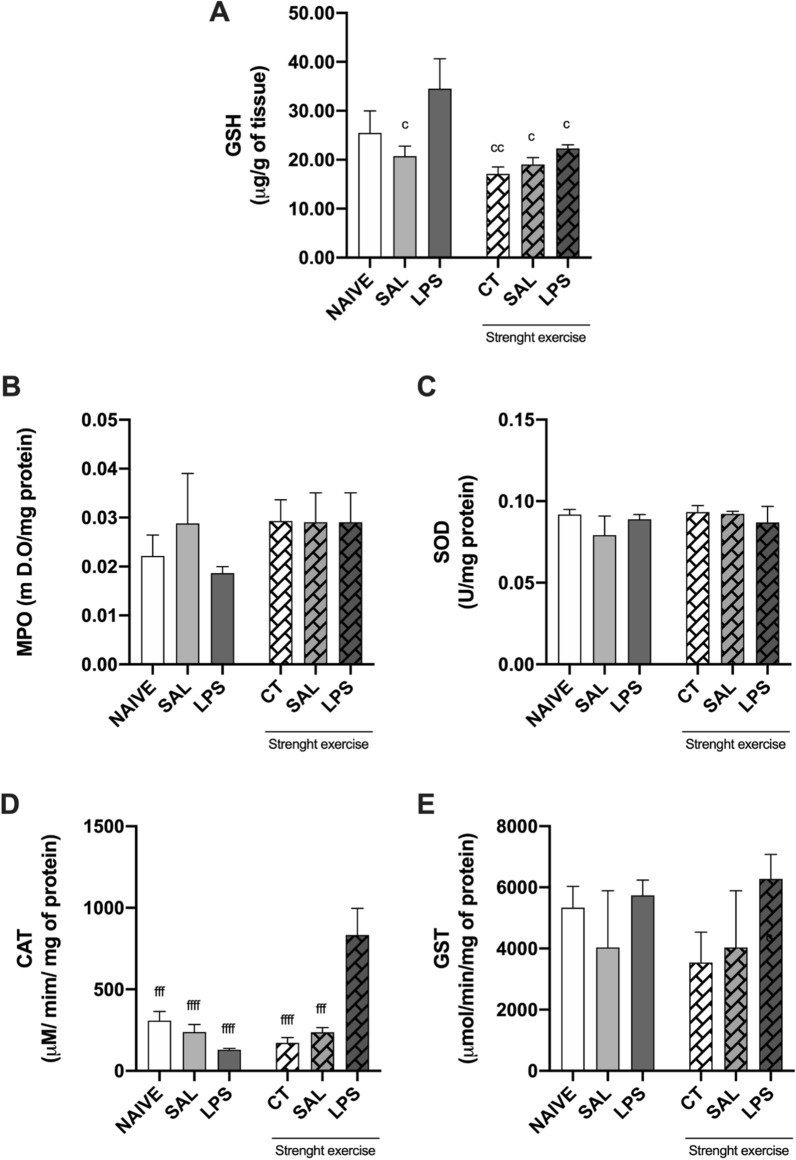
Expression of GSH and activity of the enzymes MPO, SOD, CAT and GST in the CA1 region of hippocampus of rats. **A** Eight weeks of strength exercise diminished the hippocampus concentration of GSH in the rats' hippocampus previously submitted to the strength exercise compared to the non-exercised rats, **D** increased the activity of CAT in the hippocampus of rats that received local induction of neuroinflammation, and **B**, **C** and **E** does not affect the activity of MPO, SOD and GST in the hippocampus, respectively

Figure 6A shows that the hippocampus concentration of GSH in animals that were submitted to the strength exercise was smaller than in the group that received LPS intra-hippocampal infusion when compared to the group that received only LPS intra-hippocampal infusion (p < 0.05) but was not previously submitted to the strength exercise. In its turn, Fig. 6D shows that the animals that performed strength exercise prophylactically and were submitted to the intra-hippocampal infusion of LPS have presented higher activity of the enzyme CAT in the hippocampus when compared to the group which practiced exercise and received intra-hippocampal infusion of saline, to the group that practiced only exercise and the sedentary group that received either LPS or saline (p < 0.001). The other enzymes, MPO, SOD and GST (Fig. 6B, C, and E, respectively) did not have their activities altered either by prophylactic strength exercise or by LPS intra-hippocampal infusion.

The rats were submitted to eight weeks of training in the strength exercise protocol. As followed, they received an intra-CA1 dorsal bilateral infusion of either saline (SAL) or LPS (40 µg/side). The groups NAIVE and CT were not submitted to the intra-CA1 infusion procedure. After five days of post-operative recovery, such rats were submitted to several behavioral tasks, followed by euthanasia. Then the removal of the hippocampus was performed. Results expressed in mean numbers ± EPM. (n = 7–10 per group). c P < 0.05, cc P < 0.01 compared to the non-exercised LPS group; fff P < 0.001, ffff P < 0.0001 when compared to the exercised LPS group. Statistical analysis was performed by one-way ANOVA (A: F_(5, 27)_ = 3.910; D: F_(5, 21)_ = 10.55), followed by Newman-Keuls multiple comparison test.

### Effect of strength exercise on the expression of MPO E GSH and CAT, SOD, and GST enzymes in the prefrontal cortex of rats after infusion of LPS in the CA1 region of the hippocampus

**Fig. 7:**
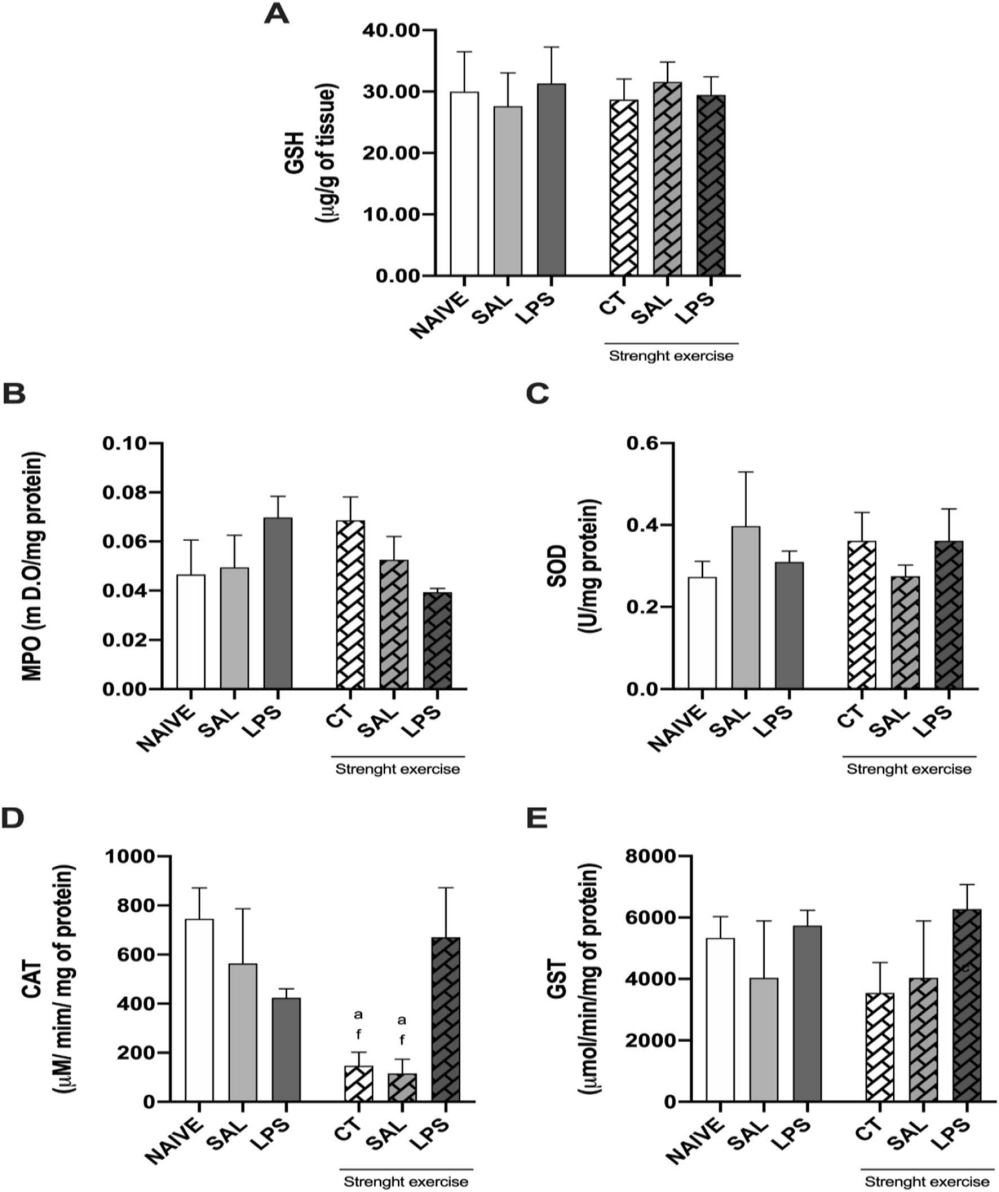
Expression of GSH and from the enzymes MPO, SOD, CAT and GST in the prefrontal cortex of rats. Eight weeks of strength exercise diminished the activity of CAT in the prefrontal cortex of rats, compared to non-exercised rats, except when the rats were submitted to intra-CA1 dorsal bilateral infusion of LPS (40 µg/side) soon after the period of exercise

As shown in Fig. 7, only the activity of CAT was diminished in animals that practiced strength exercise without the induction of intra-CA1 hippocampal neuroinflammation when compared to the group NAIVE (p < 0.05) and the group LPS exercised (p < 0.05).

The rats were submitted to eight weeks of strength exercise training protocol. As followed, they received an intra-CA1 dorsal bilateral infusion of either saline (SAL) or LPS (40 µg/side). The groups NAIVE and CT were not submitted to the intra-CA1 infusion procedure. After five days of post-operative recovery, such rats were submitted to several behavioral tasks, followed by euthanasia. Then the removal of the prefrontal cortex was performed. Panel (A-E) showing the concentration of GSH (A) and the activity of the enzymes MPO (B), SOD (C), CAT (D) and GST (E). Results expressed as mean numbers ± EPM. (n = 7–10 per group). Figure 7 (D): a P < 0.05 when compared to the group NAIVE; f P < 0.05, compared to the group LPS, which practiced strength exercise. One-way ANOVA (D: F_(5, 20)_ = 4.386) followed by Newman-Keuls multiple comparison test.

## Discussion

In this study, we investigated the influence of muscular strength exercise practiced prophylactically on oxidative stress and histological parameters and memory deficits caused by acute neuroinflammation in the hippocampus of rats. There is evidence that the practice of physical exercise increases muscle strength, promotes cardiorespiratory adaptations [33–35], influences the resolution of oxidative stress [35] and inflammatory processes [36], and presents beneficial effects on memory consolidation and retention [37].

Our results verified that the exercise promoted a gain in muscle strength assessed by the maximum load and Grip Strength Meter tests and promoted a cardiovascular adaptation assessed by the mass heart index.

Regarding histological analyses, we quantified the number of dark neurons in the hippocampus's CA1, CA3, and DG regions. Dark neurons have morphological changes, such as the presence of pyknotic nuclei and cytoplasmic reduction, as well as marked hyperbasophilia (characteristic of degenerating cells) [38]. We found that the non-exercised group that received an intra-hippocampal infusion of LPS showed a higher number of dark neurons in the CA1 region when compared to the group that received an intra-hippocampal infusion of saline, but this increase between these groups (higher number of dark neurons in the group infused intra-CA1 with LPS than with SAL) did not occur in the exercised rats. In the CA3 and DG regions, we found no significant differences between the groups.

In the oxidative stress parameters, we observed that eight weeks of muscular strength exercise prior to LPS infusion did not cause changes in the activities of the enzymes SOD, GST and MPO neither in the hippocampus nor in the prefrontal cortex of rats but caused a decrease in the concentration of the antioxidant GSH and an increase in the activity of the enzyme CAT, compared to the group that was not submitted to weeks of muscular strength exercise prior to LPS infusion.

The literature describes that the SOD enzyme is responsible for catalyzing the dismutation of superoxide anion, converting it into hydrogen peroxide, and soon afterward, the CAT or glutathione peroxidase (GPx) catalyzes the conversion of hydrogen peroxide into water and molecular oxygen [39, 40]. Therefore, we believe that the increased activity of the CAT enzyme in the animals that practiced muscular strength exercise may be due to an increase in the formation of hydrogen peroxide, thus suggesting a protective effect because, despite not being a free radical, due to the absence of unpaired electrons in the last layer, the hydrogen peroxide is an extremely deleterious ROS, capable of crossing lipidic layers and reacting with transition metals and some hemoproteins. It can also induce chromosomal alterations, break the deoxyribonucleic acid (DNA) skeleton, and oxidize sulfhydryl compounds in the absence of catalysts.

Although physical training is traditionally associated with increased concentrations of enzymatic and non-enzymatic antioxidants, the protocol of this study did not show significant influence in the resolution of oxidative stress. However, we believe that the increased concentrations of CAT enzyme may have been beneficial due to the potential decrease in tissue hydrogen peroxide concentrations, thus exerting a protective effect and may have influenced our behavioral results. However, more studies are necessary to investigate the influence of strength exercise practiced prophylactically in resolving oxidative stress.

In addition to our GSH data, Souza et al., [41] verified that a strength training protocol was able to reduce GSH levels in the hippocampus and spinal cord of rats and proposed that the modulatory effects of exercise on the glutathione system might be associated with the regulation of exercise on Nrf2/ARE mechanisms because the increased activity of enzymes that utilize GSH to scavenger ROS are subjected to regulation by Nrf2 [42].

Regarding the behavioral tests, we verified that the infusion of LPS in the CA1 region of the hippocampus of the animals caused a deficit in social and discriminative memory. However, this deficit was avoided in animals that practiced muscle strength training prophylactically, which had a better performance in short-term (SDM) and long-term recent (LTM) discriminative memory and long-term recent (LTM) social memory.

Corroborating with our results, we investigated in Araujo et al., [43] the effect of strength exercise in rats after monosodium glutamate-induced neuroinflammation. The animals received monosodium glutamate at a 4 g/kg dose from the first to the tenth day of life. After 60 days of birth, the animals started a strength exercise protocol for seven weeks and, subsequently, the object recognition test was performed. The authors found that strength exercise had an effect in decreasing the discriminative memory deficit.

According to Farzi et al., [44], beneficial effects of muscle strength exercise on discriminative memory deficit have also been reported. The authors observed that after eight weeks of muscle strength training, animals that received an intracerebroventricular injection of Aβ25-35 peptide and exercised showed a significant improvement compared to the control group regarding discriminative memory deficit (Additional file 1: Tables S1–S10).

According to Quines et al. [45], the neuroprotective effects of three models of non-pharmacological intervention were investigated: environmental enrichment, muscle strength exercise, and social enrichment in an animal model of Alzheimer's disease. The authors demonstrated that eight weeks of muscle strength training could reverse social and discriminative memory deficits after intra-hippocampal infusion of Aβ-amyloid beta-peptide in Wistar rats.

There is scientific evidence that the neuroprotective mechanisms induced by physical exercise (all modalities) are due to increased expression of brain-derived neurotrophic factor (BDNF) and insulin-like growth factor (IGF-1), neurotrophins that are involved in processes related to memory and learning. Mattson [46] suggested that since exercise increases BDNF expression in the brain, exercise may positively enhance cognitive activities, especially hippocampus-dependent activities.

Ding et al. [16] showed that physical exercise practiced for five days increased the expression of IGF-1 and BDNF in the hippocampus of rats. The authors linked such a positive influence on exercise-induced synaptic and cognitive plasticity to increased IGF-1 and BDNF in the hippocampus.

Many studies show beneficial effects of aerobic exercise in behavioral alterations facing neuroinflammation. However, studies showing the influence of muscular strength exercise are still scarce. For this reason, we found few studies showing positive effects of strength exercise on social and discriminative memory deficits. Furthermore, while the studies cited here showed the effect of strength training as a form of treatment, our study showed its preventative action.

We know that there is an awareness among the population about the beneficial effects of physical exercise. Along with this, the practice of strength exercise is increasing. In this way, research is necessary on the beneficial influence of this type of physical exercise on the human population to decrease the incidence of neurodegenerative diseases that have neuroinflammation as a background.

## Conclusion

In conclusion, the present study reinforces evidence that muscular strength exercise represents a functional prophylactic approach for preventing behavioral deficiencies and oxidative stress associated with neuroinflammation, as induced in this study by LPS. We showed the beneficial effects of strength exercise in animals that suffered local hippocampal neuroinflammation, having avoided the mnemonic deficit in tasks of discriminative and social memory, promoting the increase of the activity of the enzyme CAT in the hippocampus.

## Supplementary Information

**Additional file 1: Table S1**. Eight weeks of strength exercise previously to the infusion of intra-CA1 dorsal bilateral infusion of LPS (40 µg/side) induced an increase in the grip strength. **Table S2**. Eight weeks of strength exercise previous to the intra-CA1 dorsal bilateral infusion of LPS (40 µg/side) improves the performance of rats in the retention of the short-term (STM) or recent long-term discriminative memory (LTM) relative to the object recognition task. **Table S3**. Eight weeks of strength exercise. previously to the intra-CA1 dorsal bilateral infusion of LPS (40 µg/side). improves the performance of rats in the retention of recent long-term social memory (LTM) regarding the task of social recognition. **Table S4**. Eight weeks of strength exercise previously to the intra-CA1 dorsal bilateral infusion of (40 µg/side) induces physiological cardiac hypertrophy caused by muscular training. **Table S5**. Eight weeks of strength exercise hindered the enhancement of rats' dark neuron count verified after intra-CA1 dorsal bilateral infusion of LPS (40 µg/side) in the hippocampus CA1 region. **Table S6**. Expression of GSH and activity of the enzymes MPO. SOD. CAT and GST in the CA1 region of hippocampus of rats. (A) Eight weeks of strength exercise diminished the hippocampus concentration of GSH in the rats' hippocampus previously submitted to the strength exercise compared to the non-exercised rats. (D) increased the activity of CAT in the hippocampus of rats that received local induction of neuroinflammation. and (B. C and E) does not affect the activity of MPO. SOD and GST in the hippocampus. respectively. **Table S7**. Expression of GSH and from the enzymes MPO. SOD. CAT and GST in the prefrontal cortex of rats. Eight weeks of strength exercise diminished the activity of CAT in the prefrontal cortex of rats. compared to non-exercised rats. except when the rats were submitted to intra-CA1 dorsal bilateral infusion of LPS (40 µg/side) soon after the period of exercise. **Table S8**. Eight weeks (W) of strength exercise (three not tested weeks of adaptation plus five ones of training, which test is showed in this figure) previously to the intra-CAI dorsal bilateral infusion of LPS (40 µg/side), or saline improved the muscular strength of the animals. **Table S9**. Eight weeks of strength exercise prior to the intra-CA1 dorsal bilateral infusion of LPS (40 µg/side) does not affect rats' locomotion and exploratory activity in the open field task. **Table S10**. Eight weeks of strength exercise previously to the itra-CA1 dorsal bilateral infusion of LPS (40 µg/side) does not affect the nociception of rats in the hot plate task (DOCX 61 KB)

**Additional file 2: Figure S1**. Experimental sequence. **Figure S2**. Eight weeks of strength exercise (three not tested weeks of adaptation plus five ones of training, which test is showed in this figure) previously to the intra-CAI dorsal bilateral infusion of LPS (40 µg/side), or saline improved the muscular strength of the animals. **Figure S3**. Eight weeks of strength exercise prior to the intra-CA1 dorsal bilateral infusion of LPS (40 µg/side) does not affect rats' locomotion and exploratory activity in the open field task. **Figure S4**. Eight weeks of strength exercise previously to the intra-CA1 dorsal bilateral infusion of LPS (40 µg/side) does not affect the level of anxiety of rats in the plus-maze elevated task. **Figure S5**. Eight weeks of strength exercise previously to the itra-CA1 dorsal bilateral infusion of LPS (40 µg/side) does not affect the nociception of rats in the hot plate task. **Figure S6**. Histological analysis (image) of hippocampal regions CA1, CA3 and DG dark neurons.

## Data Availability

The datasets used or analyzed during the current study are available from the corresponding author on reasonable request.

## Abbreviations

AP: Anteroposterior
BBB: Blood brain barrier
BDNF: Brain derived neurotrophic factor
CAT: Catalase
CCL2: C–C chemokine ligand 2
CNS: Central nervous system
COX2: Cyclooxygenase 2
CT: Control group
DTNB: 5,5'-Dithio-bis-[2-nitrobenzoic acid
DV: Dorsum-ventral
ECM: Elevated crossway maze
GSH: Glutathione
GSH-Px: Glutathione-peroxidase
GSH-Rd: Glutathione-reductase
GST: S-transferase glutathione
HTAB: Hexadecyltrimethylammonium bromide
IGF-1: Insulin-like growth factor 1
LT: Lateral tilting
LTM: Long term recent memory duration
MD: Mid-lateral
MPO: Myeloperoxidase enzymes
PGC-1α: Transcriptional coactivator peroxisome proliferator-activated receptor-γ coactivator 1α
OD: Optical density
ROS: Reactive oxygen species
SOD: Superoxide-dismutase
STM: Short term memory
TMB: Tetramethylbenzene
