# Supplementary material for: Neuroprotective effects of strength training in a neuroinflammatory animal model
Source: BMC Neurosci. 2022 Apr 11;23:22. doi: 10.1186/s12868-022-00708-w (PMC8996658; doi:10.1186/s12868-022-00708-w)
Supplement: Supplementary file 1 — Additional file 1: Table S1. Eight weeks of strength exercise previously to the infusion of intra-CA1 dorsal bilateral infusion of LPS (40 µg/side) induced an increase in the grip strength. Table S2. Eight weeks of strength exercise previous to the intra-CA1 dorsal bilateral infusion of LPS (40 µg/side) improves the performance of rats in the retention of the short-term (STM) or recent long-term discriminative memory (LTM) relative to the object recognition task. Table S3. Eight weeks of strength exercise. previously to the intra-CA1 dorsal bilateral infusion of LPS (40 µg/side). improves the performance of rats in the retention of recent long-term social memory (LTM) regarding the task of social recognition. Table S4. Eight weeks of strength exercise previously to the intra-CA1 dorsal bilateral infusion of (40 µg/side) induces physiological cardiac hypertrophy caused by muscular training. Table S5. Eight weeks of strength exercise hindered the enhancement of rats' dark neuron count verified after intra-CA1 dorsal bilateral infusion of LPS (40 µg/side) in the hippocampus CA1 region. Table S6. Expression of GSH and activity of the enzymes MPO. SOD. CAT and GST in the CA1 region of hippocampus of rats. (A) Eight weeks of strength exercise diminished the hippocampus concentration of GSH in the rats' hippocampus previously submitted to the strength exercise compared to the non-exercised rats. (D) increased the activity of CAT in the hippocampus of rats that received local induction of neuroinflammation. and (B. C and E) does not affect the activity of MPO. SOD and GST in the hippocampus. respectively. Table S7. Expression of GSH and from the enzymes MPO. SOD. CAT and GST in the prefrontal cortex of rats. Eight weeks of strength exercise diminished the activity of CAT in the prefrontal cortex of rats. compared to non-exercised rats. except when the rats were submitted to intra-CA1 dorsal bilateral infusion of LPS (40 µg/side) soon after the period of exercise. Table S8. [file 12868_2022_708_MOESM1_ESM.docx]

**Raw Data**

**Tables S1.** Eight weeks of strength exercise previously to the infusion of intra-CA1 dorsal bilateral infusion of LPS (40 µg/side) induced an increase in the grip strength.

| Grip Strenght (g) | | | | | |
| --- | --- | --- | --- | --- | --- |
| Without exercise | | | Strenght exercise | | |
| NAIVE | SAL | LPS | CT | SAL | LPS |
| 950 | 1009.3 | 1289 | 1249 | 1209 | 1382 |
| 1006.6 | 974 | 1341 | 1259 | 1715 | 1225 |
| 1105.3 | 810 | 825 | 1385 | 1342 | 1731 |
| 1014 | 1065 | 1037 | 1071 | 1406 | 1301 |
| 1060 | 959 | 957 | 1044 | 1520 | 1381 |
| 793 | 763.7 | 705 | 975 | 1480 | 1052 |
| 740 | 861.3 | 633.3 | 1051 | 1098 | 1173 |
| 559 | 679.7 | 872 | 1468 | 1302 | 1101 |
| 828.7 | 626.3 | 606 | 1610 | 1242 | 1207 |
| 668.3 |  | 479 | 965 | 1313 | 1113 |

**Tables S2.** Eight weeks of strength exercise previous to the intra-CA1 dorsal bilateral infusion of LPS (40 µg/side) improves the performance of rats in the retention of the short-term (STM) or recent long-term discriminative memory (LTM) relative to the object recognition task.

A. Retention of short-term memory (STM)

| (Time exploring an object / Time exploring both objects) * 100 | | | | | | | | | | | |
| --- | --- | --- | --- | --- | --- | --- | --- | --- | --- | --- | --- |
| Without exercise | | | | | | Strenght exercise | | | | | |
| NAIVE | | SAL | | LPS | | CT | | SAL | | LPS | |
| Obj. A | Obj. C | Obj. A | Obj. C | Obj. A | Obj. C | Obj. A | Obj. C | Obj. A | Obj. C | Obj. A | Obj. C |
| 42.50 | 57.50 | 47.35 | 52.65 | 52.94 | 47.06 | 46.03 | 53.97 | 24.43 | 75.57 | 50.15 | 49.85 |
| 37.13 | 62.87 | 39.04 | 60.96 | 38.31 | 61.69 | 32.77 | 67.24 | 28.49 | 71.51 | 31.07 | 68.93 |
| 46.23 | 53.77 | 40.83 | 59.17 | 28.65 | 71.35 | 36.73 | 63.27 | 30.57 | 69.43 | 24.18 | 75.82 |
| 13.15 | 86.85 | 37.55 | 62.45 | 60.68 | 39.32 | 32.77 | 67.24 | 39.28 | 60.72 | 39.45 | 60.55 |
| 48.69 | 51.31 | 26.94 | 73.06 | 35.17 | 64.83 | 8.16 | 91.84 | 34.84 | 65.16 | 52.13 | 47.87 |
| 38.48 | 61.52 | 43.27 | 56.73 | 54.24 | 45.76 | 38.91 | 61.09 | 4.69 | 95.31 | 37.46 | 62.54 |
| 51.92 | 48.08 | 44.03 | 55.97 | 59.25 | 40.75 | 36.88 | 63.12 | 64.09 | 35.91 | 40.05 | 59.95 |
| 13.15 | 86.85 |  |  | 42.11 | 57.89 |  |  | 34.23 | 65.77 |  |  |
|  |  |  |  | 48.83 | 51.17 |  |  |  |  |  |  |
|  |  |  |  | 29.13 | 70.87 |  |  |  |  |  |  |

B. Retention of recent long-term memory (LTM)

| (Time exploring an object / Time exploring both objects) * 100 | | | | | | | | | | | |
| --- | --- | --- | --- | --- | --- | --- | --- | --- | --- | --- | --- |
| Without exercise | | | | | | Strenght exercise | | | | | |
| NAIVE | | SAL | | LPS | | CT | | SAL | | LPS | |
| Obj. A | Obj. D | Obj. A | Obj. D | Obj. A | Obj. D | Obj. A | Obj. D | Obj. A | Obj. D | Obj. A | Obj. D |
| 36.58 | 63.42 | 18.73 | 81.27 | 48.16 | 51.84 | 39.00 | 61.00 | 46.54 | 53.46 | 36.96 | 63.04 |
| 40.01 | 59.99 | 51.14 | 48.86 | 35.98 | 64.02 | 47.61 | 52.39 | 35.76 | 64.24 | 40.14 | 59.86 |
| 32.64 | 67.36 | 52.66 | 47.34 | 88.78 | 11.22 | 44.51 | 55.49 | 46.74 | 53.26 | 38.38 | 61.62 |
| 32.92 | 67.08 | 45.30 | 54.70 | 54.57 | 45.43 | 31.38 | 68.62 | 33.54 | 66.46 | 23.59 | 76.41 |
| 50.31 | 49.69 | 28.22 | 71.78 | 37.11 | 62.89 | 41.19 | 58.81 | 52.03 | 47.97 | 46.38 | 53.62 |
| 55.45 | 44.56 | 48.69 | 51.31 | 54.21 | 45.79 | 31.38 | 68.62 | 64.25 | 35.75 | 32.04 | 67.96 |
| 45.11 | 54.89 | 37.58 | 62.42 | 54.77 | 45.23 | 18.87 | 81.13 | 36.85 | 63.15 | 50.86 | 49.14 |
| 43.45 | 56.55 |  |  | 47.33 | 52.67 | 61.29 | 38.71 | 38.69 | 61.31 | 48.73 | 51.27 |
|  |  |  |  | 55.47 | 44.53 | 44.66 | 55.34 | 34.37 | 65.63 |  |  |
|  |  |  |  |  |  |  |  | 38.45 | 61.55 |  |  |

**Tables S3.** Eight weeks of strength exercise. previously to the intra-CA1 dorsal bilateral infusion of LPS (40 µg/side). improves the performance of rats in the retention of recent long-term social memory (LTM) regarding the task of social recognition.

| (Time exploring an rat / Time exploring both rats) * 100 | | | | | | | | | | | |
| --- | --- | --- | --- | --- | --- | --- | --- | --- | --- | --- | --- |
| Without exercise | | | | | | Strenght exercise | | | | | |
| NAIVE | | SAL | | LPS | | CT | | SAL | | LPS | |
| familiar | novel | familiar | novel | familiar | novel | familiar | novel | familiar | novel | familiar | novel |
| 44.09 | 55.91 | 45.89 | 54.11 | 41.21 | 58.79 | 38.10 | 61.90 | 40.47 | 59.53 | 50.00 | 50.00 |
| 43.35 | 56.65 | 46.72 | 53.28 | 47.42 | 52.58 | 42.98 | 57.02 | 50.21 | 49.79 | 48.41 | 51.59 |
| 48.29 | 51.71 | 48.54 | 51.46 | 46.19 | 53.81 | 49.21 | 50.79 | 36.01 | 63.99 | 40.91 | 59.09 |
| 50.60 | 49.40 | 38.30 | 61.70 | 62.89 | 37.11 | 49.51 | 50.49 | 39.53 | 60.47 | 31.98 | 68.02 |
| 46.61 | 53.39 | 44.34 | 55.66 | 43.69 | 56.31 | 47.31 | 52.69 | 55.77 | 44.23 | 52.43 | 47.57 |
| 48.94 | 51.06 | 46.55 | 53.45 | 58.75 | 41.25 | 44.71 | 55.29 | 52.11 | 47.89 | 45.70 | 54.30 |
|  |  | 43.78 | 56.22 | 50.00 | 50.00 |  |  | 41.44 | 58.56 | 37.36 | 62.64 |
|  |  |  |  | 54.76 | 45.24 |  |  | 30.49 | 69.51 | 38.73 | 61.27 |
|  |  |  |  | 54.41 | 45.59 |  |  | 38.14 | 61.86 | 38.14 | 61.86 |
|  |  |  |  | 42.66 | 57.34 |  |  |  |  |  |  |

**Tables S4.** Eight weeks of strength exercise previously to the intra-CA1 dorsal bilateral infusion of (40 µg/side) induces physiological cardiac hypertrophy caused by muscular training.

| Heart weight / Body weight (%) | | | | | |
| --- | --- | --- | --- | --- | --- |
| Without exercise | | | Strenght exercise | | |
| NAIVE | SAL | LPS | CT | SAL | LPS |
| 0.293 | 0.278 | 0.277 | 0.352 | 0.310 | 0.391 |
| 0.272 | 0.283 | 0.298 | 0.357 | 0.307 | 0.328 |
| 0.305 | 0.280 | 0.284 | 0.336 | 0.317 | 0.381 |
| 0.312 | 0.287 | 0.300 | 0.312 | 0.334 | 0.339 |
| 0.291 | 0.269 | 0.281 | 0.311 | 0.334 | 0.309 |
|  |  | 0.346 | 0.374 | 0.343 | 0.281 |
|  |  |  |  |  | 0.370 |

**Tables S5.** Eight weeks of strength exercise hindered the enhancement of rats' dark neuron count verified after intra-CA1 dorsal bilateral infusion of LPS (40 µg/side) in the hippocampus CA1 region.

| Dark cell count (n/mm^2^) | | | | | | | | | | | |
| --- | --- | --- | --- | --- | --- | --- | --- | --- | --- | --- | --- |
| CA1 | | | | CA3 | | | | DG | | | |
| Without exercise | | Strenght exercise | | Without exercise | | Strenght exercise | | Without exercise | | Strenght exercise | |
| SAL | LPS | SAL | LPS | SAL | LPS | SAL | LPS | SAL | LPS | SAL | LPS |
| 112.5 | 141.7 | 214.6 | 137.5 | 109.4 | 134.4 | 212.5 | 128.1 | 158.3 | 187.5 | 239.6 | 158.3 |
| 81.3 | 166.7 | 206.3 | 162.5 | 68.8 | 146.9 | 125 | 140.6 | 204.2 | 231.3 | 237.5 | 177.1 |
| 77.1 | 225.0 | 170.8 | 102.1 | 75.0 | 168.8 | 171.9 | 237.5 | 308.3 | 210.4 | 189.6 | 160.4 |
| 75.0 | 177.1 | 87.5 |  | 65.6 | 128.1 | 115 |  | 227.1 | 268.8 | 216.7 |  |

**Tables S6.** Expression of GSH and activity of the enzymes MPO. SOD. CAT and GST in the CA1 region of hippocampus of rats. (A) Eight weeks of strength exercise diminished the hippocampus concentration of GSH in the rats' hippocampus previously submitted to the strength exercise compared to the non-exercised rats. (D) increased the activity of CAT in the hippocampus of rats that received local induction of neuroinflammation. and (B. C and E) does not affect the activity of MPO. SOD and GST in the hippocampus. respectively.

A.

| GSH (μg/g of tissue) | | | | | |
| --- | --- | --- | --- | --- | --- |
| Without exercise | | | Strenght exercise | | |
| NAIVE | SAL | LPS | CT | SAL | LPS |
| 27.95 | 29.44 | 49.49 | 13.66 | 21.64 | 22.76 |
| 18.11 | 14.77 | 47.82 | 18.48 | 13.47 | 21.46 |
| 22.38 | 21.27 | 32.41 | 12.36 | 20.90 | 20.71 |
| 17.00 | 18.48 | 21.64 | 18.30 | 19.04 | 20.34 |
| 41.88 | 22.38 | 21.27 | 18.30 | 19.97 | 24.61 |
|  | 17.93 |  | 21.64 |  | 24.06 |

B.

| MPO (m D.O/mg protein) | | | | | |
| --- | --- | --- | --- | --- | --- |
| Without exercise | | | Strenght exercise | | |
| NAIVE | SAL | LPS | CT | SAL | LPS |
| 0.0204 | 0.0796 | 0.0204 | 0.0162 | 0.0214 | 0.0214 |
| 0.0123 | 0.0199 | 0.0185 | 0.0251 | 0.0205 | 0.0205 |
| 0.0359 | 0.0190 | 0.0152 | 0.0459 | 0.0514 | 0.0514 |
| 0.0147 | 0.0147 | 0.0156 | 0.0215 | 0.0198 | 0.0198 |
| 0.0273 | 0.0178 | 0.0241 | 0.0331 | 0.0322 | 0.0322 |
|  | 0.0217 | 0.0180 | 0.0341 |  |  |

C.

| SOD (U/mg protein) | | | | | |
| --- | --- | --- | --- | --- | --- |
| Without exercise | | | Strenght exercise | | |
| NAIVE | SAL | LPS | CT | SAL | LPS |
| 0.0836 | 0.0985 | 0.0852 | 0.1029 | 0.0889 | 0.0925 |
| 0.1010 | 0.0753 | 0.0902 | 0.0834 | 0.0938 | 0.1008 |
| 0.0969 | 0.0919 | 0.0822 | 0.0851 | 0.0922 | 0.0387 |
| 0.0880 | 0.0882 | 0.0905 | 0.1016 | 0.0972 | 0.0989 |
| 0.0896 | 0.0980 | 0.1039 | 0.0934 | 0.0890 | 0.1015 |
|  | 0.0236 | 0.0809 |  |  | 0.0894 |
|  |  | 0.0899 |  |  |  |

D.

| CAT (μM/min/mg of protein) | | | | | |
| --- | --- | --- | --- | --- | --- |
| Without exercise | | | Strenght exercise | | |
| NAIVE | SAL | LPS | CT | SAL | LPS |
| 241.20 | 203.78 | 108.28 | 92.86 | 171.38 | 1350.93 |
| 466.74 | 238.20 | 128.07 | 132.79 | 307.70 | 989.46 |
| 217.22 | 414.68 | 129.40 | 176.96 | 232.82 | 594.05 |
| 308.40 | 174.62 | 151.99 | 284.60 | 237.30 | 398.93 |
|  | 159.72 |  | 171.80 |  | 833.30 |

E.

| GST (μmol/min/mg of protein) | | | | | |
| --- | --- | --- | --- | --- | --- |
| Without exercise | | | Strenght exercise | | |
| NAIVE | SAL | LPS | CT | SAL | LPS |
| 6764.2 | 1175.6 | 5603.6 | 5872.8 | 1175.6 | 5960.4 |
| 3677.1 | 8998.3 | 4754.2 | 3533.8 | 8998.3 | 8050.2 |
| 4779.5 | 4749.1 | 4687.9 | 3745.5 | 4749.1 | 6842.7 |
| 6136.0 | 1234.6 | 6254.8 | 1026.0 | 1234.6 | 4246.9 |
|  |  | 7373.8 |  |  |  |

**Tables S7.** Expression of GSH and from the enzymes MPO. SOD. CAT and GST in the prefrontal cortex of rats. Eight weeks of strength exercise diminished the activity of CAT in the prefrontal cortex of rats. compared to non-exercised rats. except when the rats were submitted to intra-CA1 dorsal bilateral infusion of LPS (40 µg/side) soon after the period of exercise.

A.

| GSH (μg/g of tissue) | | | | | |
| --- | --- | --- | --- | --- | --- |
| Without exercise | | | Strenght exercise | | |
| NAIVE | SAL | LPS | CT | SAL | LPS |
| 25.73 | 22.38 | 49.49 | 32.23 | 35.75 | 27.95 |
| 48.57 | 22.94 | 47.82 | 36.50 | 42.07 | 33.53 |
| 27.21 | 22.94 | 32.41 | 37.42 | 26.84 | 40.95 |
| 18.48 | 22.57 | 15.33 | 27.40 | 28.70 | 22.01 |
|  | 54.69 | 21.64 | 17.18 | 24.61 | 21.83 |
|  | 20.16 | 21.27 | 21.08 |  | 30.37 |

B.

| MPO (m D.O/mg protein) | | | | | |
| --- | --- | --- | --- | --- | --- |
| Without exercise | | | Strenght exercise | | |
| NAIVE | SAL | LPS | CT | SAL | LPS |
| 0.0266 | 0.0168 | 0.0511 | 0.0399 | 0.0418 | 0.0365 |
| 0.0447 | 0.0765 | 0.0895 | 0.0512 | 0.0666 | 0.0381 |
| 0.0272 | 0.1074 | 0.0478 | 0.0689 | 0.0781 | 0.0433 |
| 0.1013 | 0.0132 | 0.0841 | 0.0700 | 0.0235 | 0.0425 |
| 0.0329 | 0.0268 | 0.0766 | 0.1200 | 0.0525 | 0.0365 |
|  | 0.0607 |  | 0.0617 |  |  |
|  | 0.0450 |  | 0.0686 |  |  |

C.

| SOD (U/mg protein) | | | | | |
| --- | --- | --- | --- | --- | --- |
| Without exercise | | | Strenght exercise | | |
| NAIVE | SAL | LPS | CT | SAL | LPS |
| 0.3764 | 0.3027 | 0.3606 | 0.2360 | 0.3089 | 0.2421 |
| 0.2839 | 0.2972 | 0.3435 | 0.5589 | 0.2064 | 0.4646 |
| 0.2218 | 0.9223 | 0.2924 | 0.3222 | 0.3272 | 0.2093 |
| 0.2099 | 0.2101 | 0.2418 | 0.3286 | 0.2566 | 0.2715 |
|  | 0.2544 |  |  |  | 0.6183 |

D.

| CAT (μM/min/mg of protein) | | | | | |
| --- | --- | --- | --- | --- | --- |
| Without exercise | | | Strenght exercise | | |
| NAIVE | SAL | LPS | CT | SAL | LPS |
| 497.327 | 198.789 | 398.206 | 413.593 | 46.7591 | 1065.44 |
| 840.661 | 1012.49 | 379.058 | 156.358 | 289.298 | 165.266 |
| 899.201 | 165.921 | 562.063 | 73.665 | 76.5562 | 917.274 |
|  | 879.167 | 350.766 | 47.4384 | 48.4593 | 532.489 |
|  |  | 428.973 | 102.083 |  |  |
|  |  |  | 89.5063 |  |  |

E.

| GST (μmol/min/mg of protein) | | | | | |
| --- | --- | --- | --- | --- | --- |
| Without exercise | | | Strenght exercise | | |
| NAIVE | SAL | LPS | CT | SAL | LPS |
| 6764.2 | 1175.6 | 5603.6 | 5872.8 | 1175.6 | 5960.4 |
| 3677.1 | 8998.3 | 4754.2 | 3533.8 | 8998.3 | 8050.2 |
| 4779.5 | 4749.1 | 4687.9 | 3745.5 | 4749.1 | 6842.7 |
| 6136.0 | 1234.6 | 6254.8 | 1026.0 | 1234.6 | 4246.9 |
|  |  | 7373.8 |  |  |  |

**Tables S8.** Eight weeks (W) of strength exercise (three not tested weeks of adaptation plus five ones of training, which test is showed in this figure) previously to the intra-CAI dorsal bilateral infusion of LPS (40 µg/side), or saline improved the muscular strength of the animals.

| Strenght gain (g) | | | | | | | | | | | | | | |
| --- | --- | --- | --- | --- | --- | --- | --- | --- | --- | --- | --- | --- | --- | --- |
| CT | | | | | SAL | | | | | LPS | | | | |
| W4 | W5 | W6 | W7 | W8 | W4 | W5 | W6 | W7 | W8 | W4 | W5 | W6 | W7 | W8 |
| 525 | 431 | 419 | 509 | 541 | 473 | 363 | 400 | 406 | 383 | 335 | 390 | 437 | 472 | 483 |
| 453 | 472 | 514 | 522 | 603 | 520 | 521 | 586 | 583 | 603 | 396 | 443 | 559 | 708 | 762 |
| 504 | 404 | 477 | 456 | 549 | 467 | 507 | 546 | 502 | 534 | 490 | 443 | 528 | 522 | 617 |
| 413 | 480 | 515 | 559 | 613 | 421 | 519 | 570 | 572 | 620 | 507 | 472 | 592 | 469 | 567 |
| 386 | 398 | 491 | 541 | 535 | 622 | 554 | 617 | 590 | 601 | 431 | 526 | 487 | 400 | 503 |
| 307 | 291 | 258 | 347 | 367 | 378 | 415 | 511 | 507 | 604 | 357 | 384 | 466 | 508 | 456 |
| 400 | 463 | 536 | 532 | 581 | 444 | 483 | 383 | 382 | 416 | 436 | 426 | 454 | 523 | 479 |
| 412 | 392 | 442 | 554 | 587 | 349 | 347 | 384 | 426 | 491 | 409 | 539 | 439 | 511 | 492 |
| 302 | 361 | 506 | 481 | 527 | 400 | 437 | 430 | 458 | 477 | 389 | 347 | 512 | 578 | 654 |
| 367 | 473 | 492 | 571 | 560 | 368 | 391 | 434 | 397 | 450 | 414 | 457 | 475 | 540 | 591 |

**Tables S9.** Eight weeks of strength exercise prior to the intra-CA1 dorsal bilateral infusion of LPS (40 µg/side) does not affect rats' locomotion and exploratory activity in the open field task.

A.

| Crossings | | | | | | | | | | | |
| --- | --- | --- | --- | --- | --- | --- | --- | --- | --- | --- | --- |
| Without exercise | | | | | | | | | | | |
| NAIVE | | | | SAL | | | | LPS | | | |
| D1 | D2 | D3 | D4 | D1 | D2 | D3 | D4 | D1 | D2 | D3 | D4 |
| 52 | 68 | 64 | 63 | 72 | 54 | 65 | 35 | 1 | 28 | 36 | 57 |
| 74 | 92 | 113 | 86 | 56 | 95 | 80 | 59 | 6 | 31 | 11 | 51 |
| 92 | 66 | 60 | 68 | 4 | 34 | 51 | 66 | 46 | 102 | 70 | 42 |
| 90 | 69 | 59 | 67 | 10 | 46 | 36 | 51 | 69 | 0 | 53 | 75 |
| 16 | 42 | 48 | 25 | 18 | 45 | 27 | 24 | 69 | 46 | 62 | 89 |
| 33 | 8 | 42 | 0 | 36 | 29 | 54 | 63 | 59 | 26 | 24 | 46 |
| 61 | 63 | 49 | 65 | 37 | 15 | 24 | 20 | 75 | 74 | 56 | 81 |
| 91 | 70 | 82 | 115 | 64 | 60 | 43 | 48 | 33 | 44 | 59 | 60 |
| 32 | 20 | 26 | 62 | 100 | 42 | 37 | 74 | 15 | 35 | 14 | 56 |
|  |  |  |  |  |  |  |  | 42 | 47 | 73 | 58 |
| Strenght exercise | | | | | | | | | | | |
| CT | | | | SAL | | | | LPS | | | |
| D1 | D2 | D3 | D4 | D1 | D2 | D3 | D4 | D1 | D2 | D3 | D4 |
| 43 | 95 | 91 | 76 | 52 | 79 | 92 | 88 | 62 | 66 | 59 | 43 |
| 42 | 53 | 24 | 64 | 79 | 105 | 109 | 123 | 74 | 70 | 68 | 74 |
| 19 | 22 | 70 | 35 | 80 | 80 | 101 | 93 | 50 | 54 | 32 | 61 |
| 77 | 30 | 24 | 14 | 81 | 102 | 124 | 92 | 40 | 21 | 18 | 36 |
| 45 | 67 | 73 | 55 | 12 | 11 | 24 | 23 | 70 | 50 | 80 | 30 |
| 73 | 77 | 72 | 61 | 46 | 47 | 38 | 64 | 15 | 28 | 31 | 24 |
| 25 | 15 | 38 | 53 | 79 | 75 | 71 | 53 | 71 | 54 | 47 | 44 |
| 88 | 74 | 50 | 81 | 71 | 88 | 134 | 81 | 54 | 61 | 54 | 32 |
|  |  |  |  | 49 | 32 | 44 | 47 | 37 | 20 | 48 | 47 |
|  |  |  |  | 30 | 45 | 50 | 28 |  |  |  |  |

B.

| Rearings | | | | | | | | | | | |
| --- | --- | --- | --- | --- | --- | --- | --- | --- | --- | --- | --- |
| Without exercise | | | | | | | | | | | |
| NAIVE | | | | SAL | | | | LPS | | | |
| D1 | D2 | D3 | D4 | D1 | D2 | D3 | D4 | D1 | D2 | D3 | D4 |
| 24 | 30 | 23 | 27 | 24 | 22 | 25 | 7 | 3 | 10 | 12 | 20 |
| 25 | 43 | 47 | 34 | 30 | 26 | 37 | 12 | 8 | 20 | 5 | 26 |
| 26 | 20 | 23 | 20 | 4 | 14 | 19 | 20 | 32 | 16 | 21 | 12 |
| 44 | 22 | 16 | 19 | 1 | 14 |  | 13 | 34 | 15 | 24 | 26 |
| 11 | 7 | 15 | 10 | 10 | 8 | 10 | 7 | 31 | 14 | 24 | 25 |
| 17 | 4 | 7 | 1 | 12 | 7 | 11 | 9 | 12 | 8 | 5 | 16 |
| 27 | 21 | 18 | 21 | 17 | 5 | 7 | 7 | 34 | 24 | 22 | 29 |
| 53 | 43 | 33 | 38 | 36 | 12 | 21 | 26 | 13 | 21 | 23 | 23 |
| 16 | 8 | 11 | 15 | 38 | 10 | 14 | 28 | 12 | 10 | 11 | 16 |
|  |  |  |  |  |  |  |  | 16 | 13 | 23 | 16 |
| Strenght exercise | | | | | | | | | | | |
| CT | | | | SAL | | | | LPS | | | |
| D1 | D2 | D3 | D4 | D1 | D2 | D3 | D4 | D1 | D2 | D3 | D4 |
| 9 | 44 | 23 | 25 | 24 | 51 | 59 | 41 | 23 | 33 | 25 | 23 |
| 36 | 19 | 16 | 20 | 23 | 35 | 37 | 53 | 27 | 37 | 35 | 42 |
| 17 | 14 | 9 | 19 | 40 | 23 | 26 | 25 | 29 | 19 | 16 | 12 |
| 28 | 25 | 10 | 4 | 35 | 33 | 37 | 30 | 22 | 14 | 10 | 6 |
| 18 | 18 | 26 | 26 | 2 | 8 | 4 | 3 | 41 | 11 | 15 | 7 |
| 26 | 21 | 16 | 25 | 33 | 20 | 11 | 20 | 7 | 10 | 4 | 6 |
| 11 | 5 | 13 | 11 | 28 | 23 | 21 | 18 | 38 | 36 | 23 | 16 |
| 37 | 22 | 11 | 26 | 23 | 26 | 30 | 19 | 19 | 18 | 11 | 11 |
|  |  |  |  | 12 | 18 | 14 | 11 | 29 | 12 | 14 | 9 |
|  |  |  |  | 18 | 10 | 9 | 7 |  |  |  |  |

C.

| Faecal bolus | | | | | | | | | | | |
| --- | --- | --- | --- | --- | --- | --- | --- | --- | --- | --- | --- |
| Without exercise | | | | | | | | | | | |
| NAIVE | | | | SAL | | | | LPS | | | |
| D1 | D2 | D3 | D4 | D1 | D2 | D3 | D4 | D1 | D2 | D3 | D4 |
| 1 | 1 | 0 | 0 | 0 | 0 | 0 | 0 | 0 | 0 | 0 | 0 |
| 0 | 0 | 0 | 0 | 0 | 0 | 0 | 0 | 0 | 2 | 2 | 1 |
| 0 | 0 | 0 | 1 | 0 | 0 | 0 | 0 | 0 | 0 | 0 | 3 |
| 0 | 0 | 0 | 0 | 2 | 0 | 0 | 0 | 4 | 0 | 0 | 0 |
| 0 | 0 | 0 | 0 | 3 | 2 | 1 | 0 | 1 | 0 | 0 | 0 |
| 3 | 3 | 2 | 1 | 3 | 1 | 0 | 1 | 0 | 1 | 1 | 0 |
| 4 | 3 | 0 | 1 | 3 | 4 | 2 | 2 | 0 | 0 | 0 | 0 |
| 0 | 0 | 0 | 0 | 3 | 2 | 1 | 0 | 2 | 0 | 0 | 0 |
| 3 | 2 | 2 | 2 | 0 | 0 | 0 | 0 | 5 | 2 | 0 | 1 |
|  |  |  |  |  |  |  |  | 1 | 3 | 0 | 0 |
| Strenght exercise | | | | | | | | | | | |
| CT | | | | SAL | | | | LPS | | | |
| D1 | D2 | D3 | D4 | D1 | D2 | D3 | D4 | D1 | D2 | D3 | D4 |
| 1 | 1 | 0 | 1 | 2 | 1 | 0 | 0 | 2 | 3 | 1 | 1 |
| 6 | 2 | 2 | 3 | 0 | 0 | 0 | 0 | 3 | 0 | 0 | 0 |
| 4 | 2 | 1 | 2 | 1 | 2 | 1 | 0 | 1 | 1 | 0 | 4 |
| 2 | 5 | 3 | 0 | 0 | 0 | 0 | 0 | 3 | 4 | 0 | 1 |
| 3 | 3 | 3 | 4 | 0 | 0 | 0 | 0 | 0 | 3 | 0 | 2 |
| 0 | 0 | 0 | 0 | 5 | 0 | 0 | 0 | 0 | 2 | 2 | 1 |
| 1 | 0 | 0 | 0 | 1 | 2 | 4 | 2 | 2 | 2 | 1 | 0 |
| 0 | 0 | 0 | 0 | 0 | 2 | 1 | 1 | 2 | 1 | 1 | 1 |
|  |  |  |  | 0 | 1 | 0 | 1 | 2 | 0 | 1 | 1 |
|  |  |  |  | 2 | 6 | 1 | 1 |  |  |  |  |

**Figure S3.** Eight weeks of strength exercise previously to the intra-CA1 dorsal bilateral infusion of LPS (40 µg/side) does not affect the level of anxiety of rats in the plus-maze elevated task.

A.

| Open arm entries | | | | | |
| --- | --- | --- | --- | --- | --- |
| Without exercise | | | Strenght exercise | | |
| NAIVE | SAL | LPS | CT | SAL | LPS |
| 7 | 6 | 4 | 10 | 5 | 5 |
| 3 | 5 | 5 | 8 | 7 | 6 |
| 6 | 4 | 1 | 2 | 12 | 5 |
| 8 | 7 | 8 | 4 | 12 | 3 |
| 5 | 1 | 6 | 6 | 5 | 1 |
| 4 | 10 | 5 | 2 | 4 | 6 |
| 9 | 6 | 9 | 5 | 3 | 6 |
| 8 | 5 | 4 | 4 | 7 | 6 |
| 5 | 7 | 4 | 4 | 4 | 7 |
|  |  |  |  | 6 |  |

B.

| Closed arm entries | | | | | |
| --- | --- | --- | --- | --- | --- |
| Without exercise | | | Strenght exercise | | |
| NAIVE | SAL | LPS | CT | SAL | LPS |
| 5 | 7 | 6 | 9 | 5 | 6 |
| 6 | 8 | 5 | 10 | 6 | 7 |
| 9 | 10 | 13 | 7 | 5 | 3 |
| 5 | 4 | 4 | 5 | 10 | 7 |
| 6 | 3 | 7 | 6 | 4 | 3 |
| 4 | 7 | 6 | 9 | 5 | 7 |
| 7 | 7 | 9 | 5 | 9 | 10 |
| 8 | 5 | 5 | 9 | 10 | 8 |
| 4 | 6 | 5 | 9 | 8 | 5 |
|  |  |  |  | 7 |  |

C.

| Time spent in open arms (s) | | | | | |
| --- | --- | --- | --- | --- | --- |
| Without exercise | | | Strenght exercise | | |
| NAIVE | SAL | LPS | CT | SAL | LPS |
| 137,4 | 126 | 126 | 147 | 92,4 | 134,4 |
| 120,6 | 120,6 | 140,4 | 88,2 | 147 | 139,6 |
| 124,2 | 78 | 30 | 15,21 | 189,6 | 252,6 |
| 189 | 189 | 198 | 69,19 | 147,6 | 55,21 |
| 78 | 15,4 | 141,6 | 186,85 | 126,62 | 39,97 |
| 79,2 | 142,2 | 92,4 | 53 | 174,2 | 82,02 |
| 183 | 95,4 | 187,2 | 150,6 | 55,15 | 89,84 |
| 132 | 90 | 69,6 | 79,06 | 133,25 | 135,64 |
| 212,4 | 133,8 | 127,2 | 128,62 | 79,69 | 132,93 |
|  |  |  |  | 111,73 |  |

D.

| Time spent in closed arms (s) | | | | | |
| --- | --- | --- | --- | --- | --- |
| Without exercise | | | Strenght exercise | | |
| NAIVE | SAL | LPS | CT | SAL | LPS |
| 82,8 | 139,2 | 168 | 137,4 | 132 | 131,4 |
| 151,2 | 130,2 | 86,4 | 186 | 93,6 | 123,6 |
| 137,4 | 184,8 | 139,4 | 216,28 | 66 | 27 |
| 78 | 76,2 | 61,8 | 179,27 | 89,4 | 193,1 |
| 142,2 | 206,4 | 129 | 89,5 | 169,06 | 219,09 |
| 153,6 | 87,6 | 148,8 | 184,2 | 160,37 | 105,15 |
| 75,26 | 145,8 | 81,6 | 215 | 227,56 | 185,74 |
| 128,4 | 181,2 | 187,8 | 188,13 | 141,77 | 143,37 |
| 55,52 | 87,6 | 146,4 | 142,2 | 197,42 | 152,6 |
|  |  |  |  | 156 |  |

**Tables S10.** Eight weeks of strength exercise previously to the itra-CA1 dorsal bilateral infusion of LPS (40 µg/side) does not affect the nociception of rats in the hot plate task.

| Paws withdraw latency (s) | | | | | |
| --- | --- | --- | --- | --- | --- |
| Without exercise | | | Strenght exercise | | |
| NAÏVE | SAL | LPS | CT | SAL | LPS |
| 8,85 | 4,25 | 4,68 | 3,06 | 7,12 | 13,59 |
| 3,89 | 4,69 | 4,44 | 2,15 | 6,5 | 4,18 |
| 4,12 | 6,4 | 9,13 | 4,94 | 5,04 | 6,69 |
| 2,04 | 5,63 | 3,43 | 6 | 8 | 4,66 |
| 3,46 | 5,5 | 4,06 | 6,37 | 4,82 | 5,22 |
| 4,32 | 7,66 | 6,31 | 5,59 | 4,81 | 6,7 |
| 6,97 | 7 | 3,91 | 6,43 | 6,85 | 5,74 |
| 5,07 | 8,12 | 5,81 | 5,47 | 4,63 | 4,15 |
| 7,97 | 7,16 | 3,95 | 2,94 | 8,94 | 4,78 |
|  |  | 7,13 |  | 9,16 |  |
