# Supplementary material for: Neuroprotective effects of strength training in a neuroinflammatory animal model
Source: BMC Neurosci. 2022 Apr 11;23:22. doi: 10.1186/s12868-022-00708-w (PMC8996658; doi:10.1186/s12868-022-00708-w)
Supplement: Supplementary file 2 — Additional file 2: Figure S1. Experimental sequence. Figure S2. Eight weeks of strength exercise (three not tested weeks of adaptation plus five ones of training, which test is showed in this figure) previously to the intra-CAI dorsal bilateral infusion of LPS (40 µg/side), or saline improved the muscular strength of the animals. Figure S3. Eight weeks of strength exercise prior to the intra-CA1 dorsal bilateral infusion of LPS (40 µg/side) does not affect rats' locomotion and exploratory activity in the open field task. Figure S4. Eight weeks of strength exercise previously to the intra-CA1 dorsal bilateral infusion of LPS (40 µg/side) does not affect the level of anxiety of rats in the plus-maze elevated task. Figure S5. Eight weeks of strength exercise previously to the itra-CA1 dorsal bilateral infusion of LPS (40 µg/side) does not affect the nociception of rats in the hot plate task. Figure S6. Histological analysis (image) of hippocampal regions CA1, CA3 and DG dark neurons. [file 12868_2022_708_MOESM2_ESM.docx]

**Supplementary Material**

**Figure S1.** **Experimental sequence**


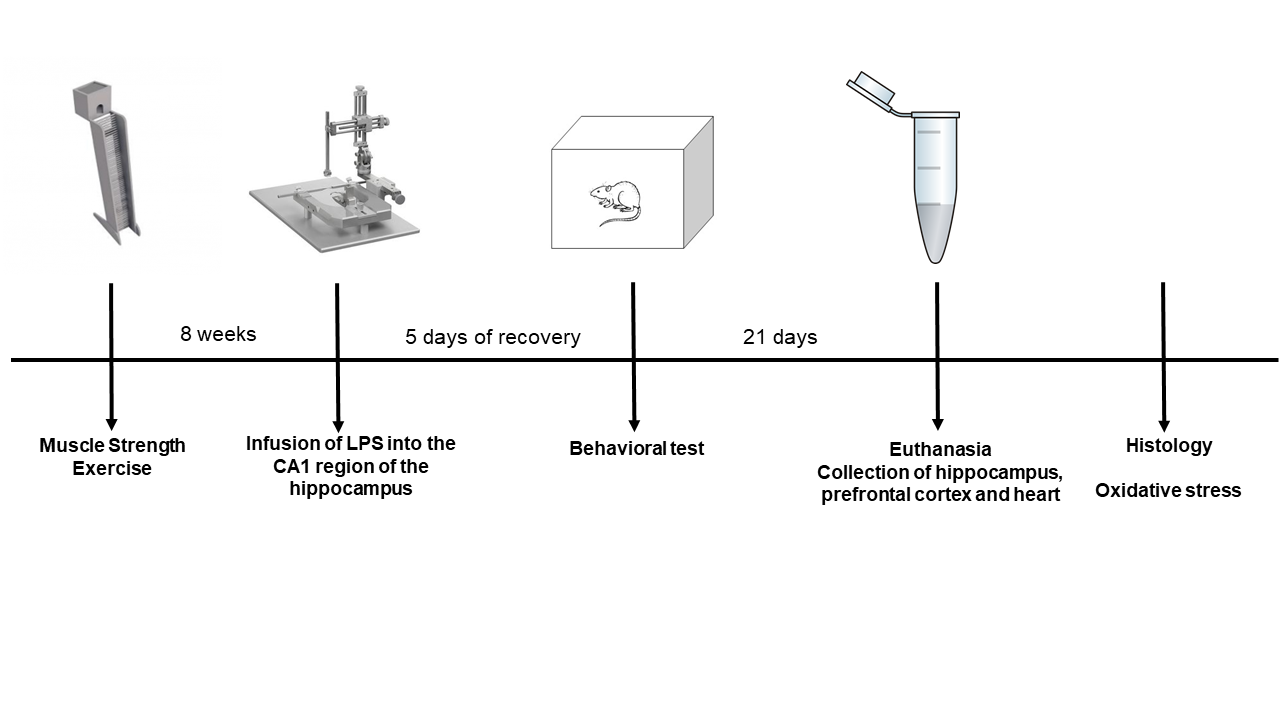


Initially, an eight-week muscle strength exercise protocol was performed. Then, LPS or saline infusion was performed in the CA1 region of the hippocampus through stereotaxic surgery. After surgery, the animals were in recovery for five days. Afterward, the animals performed the behavioral tests described in the methodology for three weeks (21 days). Finally, the animals were euthanized, and the hippocampus and prefrontal cortex were collected for analysis.

**Figure S2.** Eight weeks of strength exercise (three not tested weeks of adaptation plus five ones of training, which test is showed in this figure) previously to the intra-CAI dorsal bilateral infusion of LPS (40 µg/side), or saline improved the muscular strength of the animals.


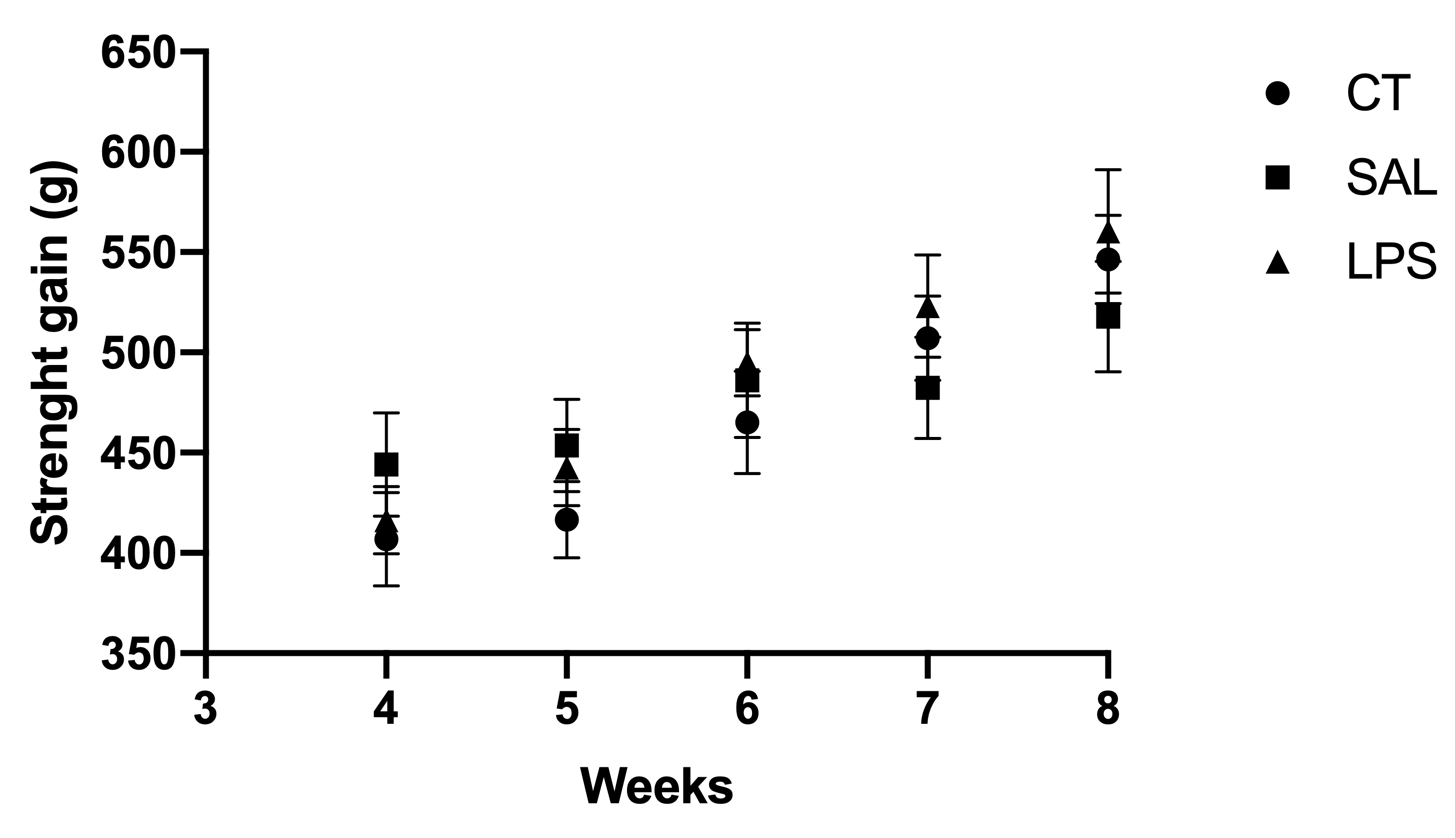


The animals were submitted to the maximum load test once a week during all the muscular strength training protocols. The animals achieved gradual muscular gain along days (two-way ANOVA, F_(2.395, 64.67)_ = 27.87, p < 0.0001). However, there were no statistical differences among the groups (two-way ANOVA, F_(2, 27)_ = 0.2573, p = 0.7750, followed by the Newman-Keuls multiple comparison test).

**Figure S3.** Eight weeks of strength exercise prior to the intra-CA1 dorsal bilateral infusion of LPS (40 µg/side) does not affect rats' locomotion and exploratory activity in the open field task.


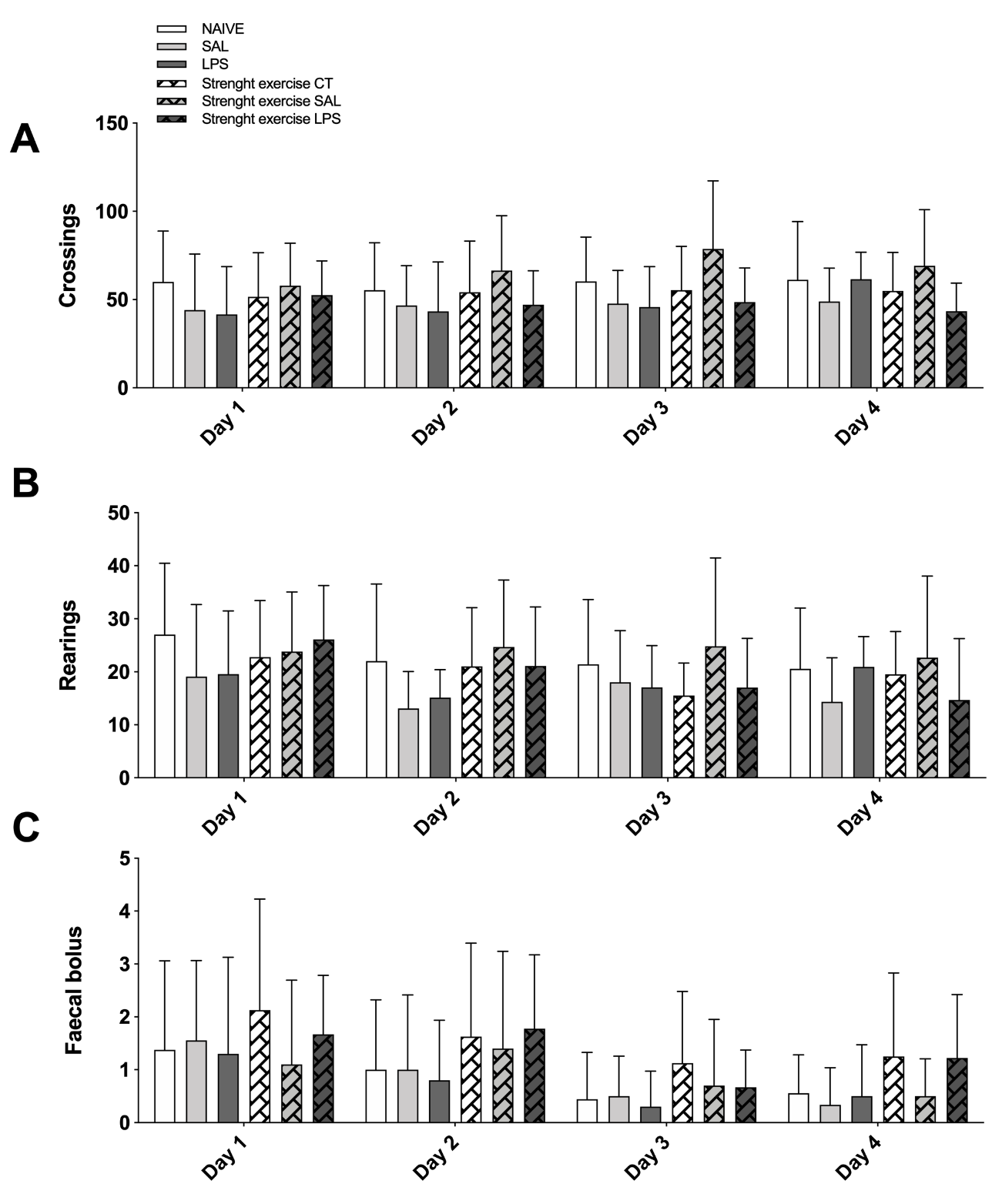


The rats were submitted to two weeks of training in the strength exercise protocol. As followed, they received an intra-CA1 dorsal bilateral infusion of either saline (SAL) or LPS (40 µg/side). The groups NAIVE and control (CT) were not submitted to the procedure of intra-CA1 infusion. After five days of post-operative recovery, these rats were submitted to several behavioral tasks, starting with the open-field task, as part of the habituation process that preceded the object recognition task. Four sessions were performed, one by day, during four consecutive days. (A) Number of crossings among internal squares of the open field box. (B) Number of times that the rat lifted its back legs. (C) Number of feces expelled by the rat during the period of session in open-field. Data are expressed as mean numbers (± EM) from the previous variables. There was no significant statistical difference among the groups after two-way ANOVA with repeated measurements, followed by the Newman-Keuls multiple comparison test.

**Figure S4.** Eight weeks of strength exercise previously to the intra-CA1 dorsal bilateral infusion of LPS (40 µg/side) does not affect the level of anxiety of rats in the plus-maze elevated task.


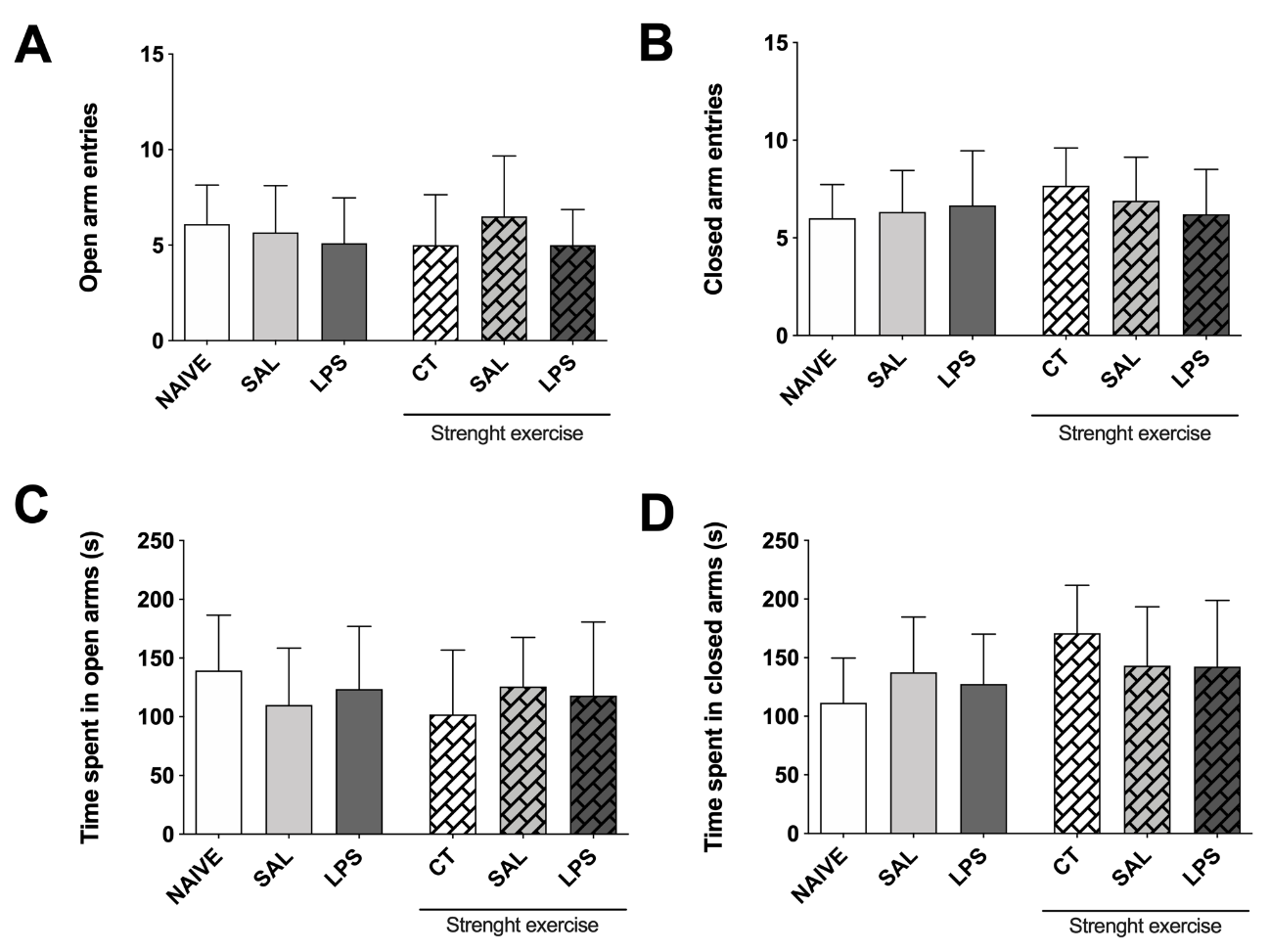


The rats were submitted to the strength exercise for two weeks of the training protocol. As followed, they received an intra-CA1 dorsal bilateral infusion of saline (SAL) or LPS (40 µg/side). The groups NAIVE and control (CT) were not submitted to the intra-CA1 infusion procedure. After five days of post-operative recovery, these rats were submitted to several behavioral tasks, including the elevated plus-maze task. (A) Number of entries in the open arms. (B) Number of entries in the closed arms. (C) Time spent within the open arms. (D) Time spent within the closed arms. Data are expressed as mean numbers (± EM) from previous variables. There was no significant difference among the groups, either after one-way ANOVA followed by multiple comparison Student-Newman-Keuls test, or after comparisons with the NAIVE group, according to the test of Dunnett.

**Figure S5.** Eight weeks of strength exercise previously to the itra-CA1 dorsal bilateral infusion of LPS (40 µg/side) does not affect the nociception of rats in the hot plate task.


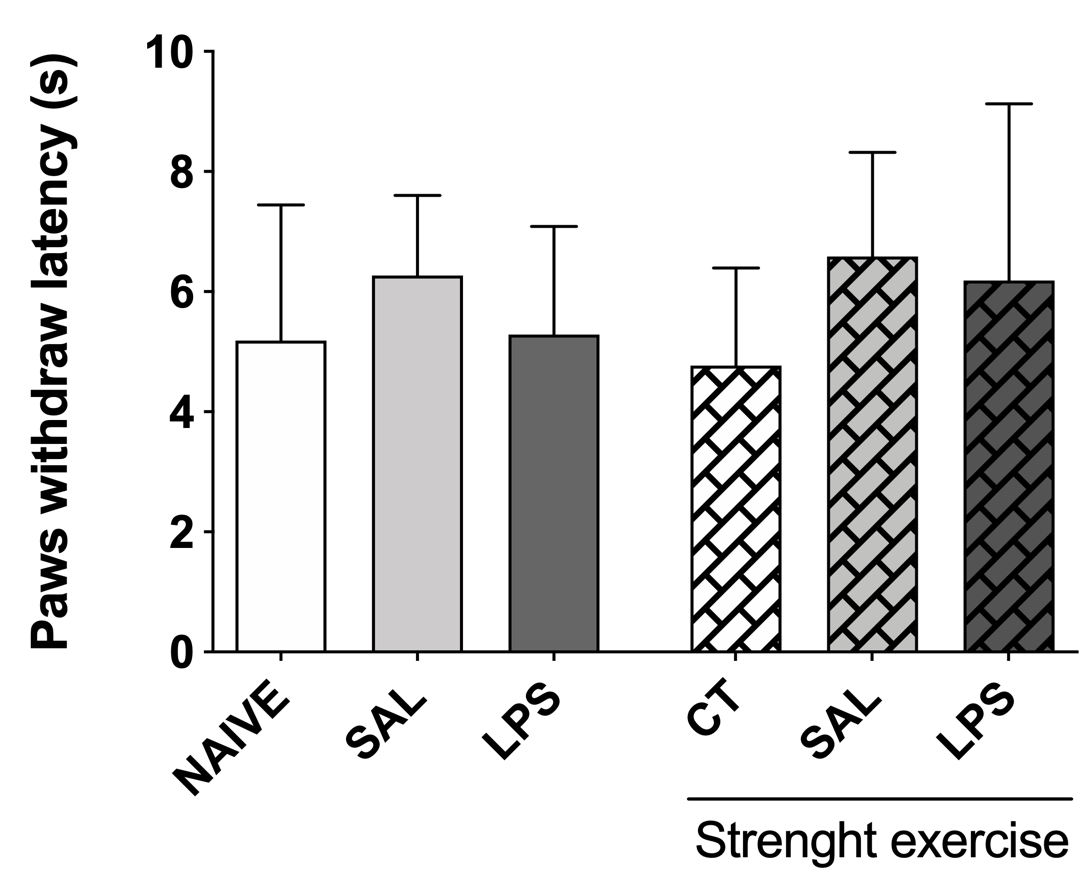


The rats were submitted to eight weeks of training in the strength exercise protocol. As followed, they received the intra-CA1 dorsal bilateral infusion of saline (SAL) or LPS (40 µg/side). The groups NAIVE and control (CT) were not submitted to the intra-CA1 infusion procedure. After five days of post-operative recovery, these rats were submitted to several behavioral activities, including the hot plate task. The data are expressed as mean numbers (± EM) of the reaction time to the thermal stimulation (removal and lick of the legs). There was no statistically significant difference among the groups, either after one-way ANOVA followed by the Student-Newman-Keuls multiple comparison test, or after the comparisons with the NAIVE group, according to the Dunnett’s test.

**Figure S6.** Histological analysis (image) of hippocampal regions CA1, CA3 and DG dark neurons.


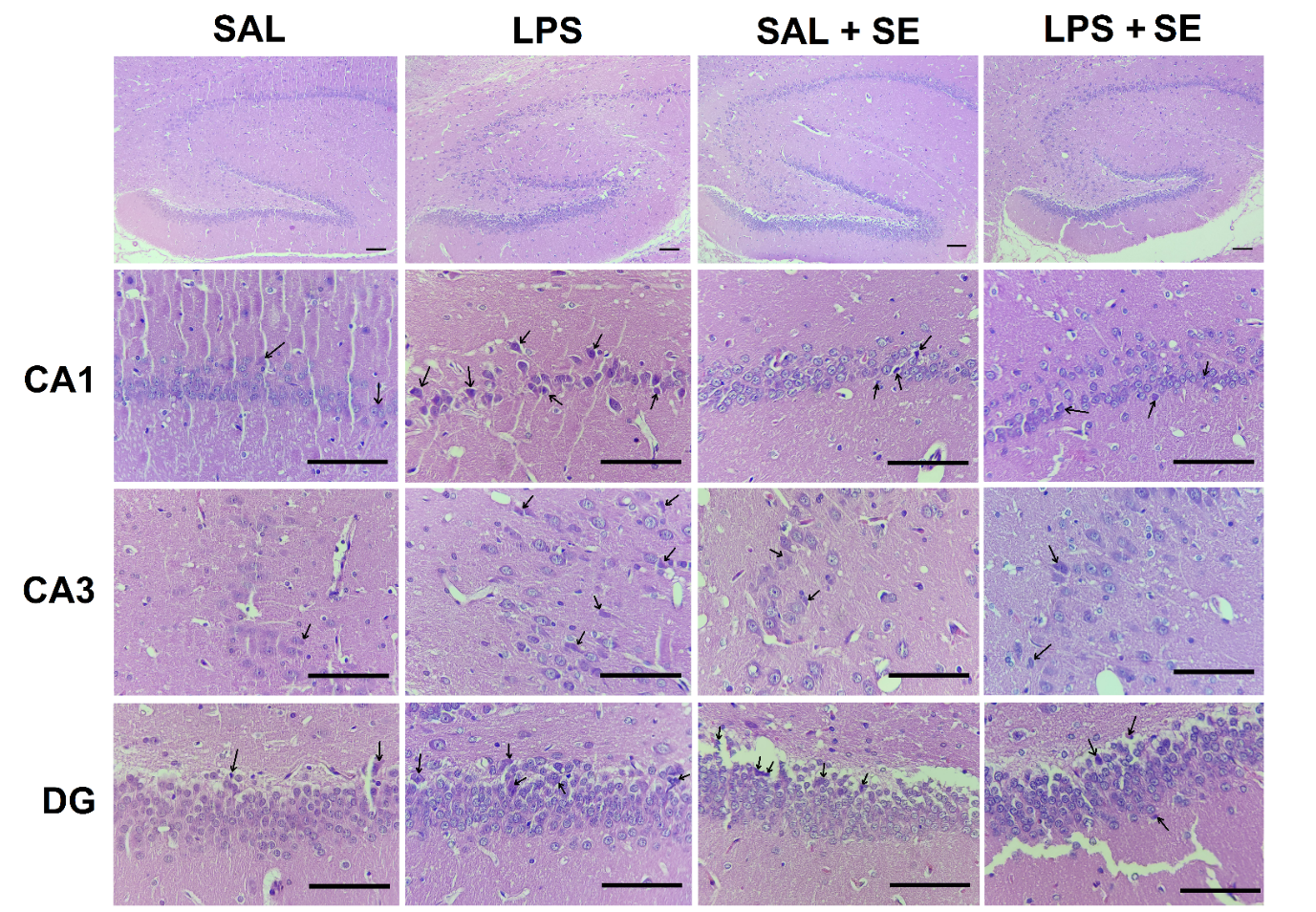


The rats were submitted to eight weeks of training in the strength exercise (SE) protocol. As followed, they received the intra-CA1 dorsal bilateral infusion of saline (SAL + SE) or LPS (LPS + SE) (40 µg/side). The groups control (SAL) e (LPS) not practice muscle strength exercise. Dark neurons (indicated by arrows) were recognized by hyperbasophilia, reduced cytoplasm and morphological changes. Scale bar represents 200μm.
